# Characterising the RNA-binding protein atlas of the mammalian brain uncovers RBM5 misregulation in mouse models of Huntington’s disease

**DOI:** 10.1038/s41467-023-39936-x

**Published:** 2023-07-19

**Authors:** Meeli Mullari, Nicolas Fossat, Niels H. Skotte, Andrea Asenjo-Martinez, David T. Humphreys, Jens Bukh, Agnete Kirkeby, Troels K. H. Scheel, Michael L. Nielsen

**Affiliations:** 1 Proteomics Program, Novo Nordisk Foundation Center for Protein Research, Faculty of Health and Medical Sciences, University of Copenhagen, Copenhagen, Denmark; 2 Copenhagen Hepatitis C Program (CO-HEP), Department of Immunology and Microbiology, Faculty of Health and Medical Sciences, University of Copenhagen, Copenhagen, Denmark; 3 CO-HEP, Department of Infectious Diseases, Copenhagen University Hospital, Hvidovre, Denmark; 4 Department of Drug Design and Pharmacology, Faculty of Health and Medical Sciences, University of Copenhagen, Copenhagen, Denmark; 5 The Novo Nordisk Foundation Center for Stem Cell Medicine (reNEW) and Department of Neuroscience, University of Copenhagen, Copenhagen, Denmark; 6 Victor Chang Cardiac Research Institute, Darlinghurst, NSW 2010 Australia; 7 Wallenberg Center for Molecular Medicine (WCMM) and Department of Experimental Medical Science, Lund University, Lund, Sweden; 8 Laboratory of Virology and Infectious Disease, The Rockefeller University, New York, NY USA

**Keywords:** Proteomics, Huntington's disease, RNA-binding proteins

## Abstract

RNA-binding proteins (RBPs) are key players regulating RNA processing and are associated with disorders ranging from cancer to neurodegeneration. Here, we present a proteomics workflow for large-scale identification of RBPs and their RNA-binding regions in the mammalian brain identifying 526 RBPs. Analysing brain tissue from males of the Huntington’s disease (HD) R6/2 mouse model uncovered differential RNA-binding of the alternative splicing regulator RBM5. Combining several omics workflows, we show that RBM5 binds differentially to transcripts enriched in pathways of neurodegeneration in R6/2 brain tissue. We further find these transcripts to undergo changes in splicing and demonstrate that RBM5 directly regulates these changes in human neurons derived from embryonic stem cells. Finally, we reveal that RBM5 interacts differently with several known huntingtin interactors and components of huntingtin aggregates. Collectively, we demonstrate the applicability of our method for capturing RNA interactor dynamics in the contexts of tissue and disease.

## Abstract (web summary)

RNA-Binding Proteins (RBPs) are critical regulators of RNA biology. Here, the authors describe the Brain-pCLAP methodology, uncover the RBP atlas of the mouse brain and demonstrate the differential binding of the splicing factor RBM5 to Huntington’s disease relevant transcripts in R6/2 mice.

## Introduction

RNA-binding proteins (RBPs) are key regulators of gene expression, controlling a plethora of RNA processing events such as capping, splicing, editing, transport, translation, degradation and storage^1,2^. Altogether, RBPs and RNAs constitute a sophisticated network of dynamic ribonucleoprotein particles (RNPs), where each RBP is engaged in specific step(s) of the RNA-processing pathway^3^. Alterations of the RNA-binding properties of RBPs affect RNP formation and RNA processing, and can result in disorders ranging from cancer to neurodegenerative diseases^4^.

In the brain, RNA processing is necessary for neurogenesis, differentiation, synaptic plasticity and other neuronal-specific functions^5–7^. Alternative splicing occurs at a significantly higher rate in the brain compared to other tissues^8–10^ and has determining roles in neuronal development, neuronal activity and for the functions of neuronal proteins^7,11^. Likewise, neuronal activation dependent RNA localization and translation underlie synaptic plasticity, memory, axon guidance and regeneration^12,13^. Altogether, the brain hosts one of the most abundant and diverse RNA landscapes^14^, an observation that must be supported by the existence of complex post-transcriptional regulation mechanisms. Accordingly, many RBPs have higher expression levels in the brain compared to other tissues^9^, specific expression patterns^15^ and exert brain specific functions; e.g. during transport to axons and dendrites, where RBPs protect mRNAs from degradation and premature translation^6,16^.

Beyond this, mutations in specific RBP genes, such as *FMR1* in fragile X syndrome and *PABPN1* in oculopharyngeal muscular dystrophy, have been linked to several neurological and neurodegenerative diseases^4,17–19^. Huntington’s disease (HD) manifests in atrophy of the striatum, involuntary movements, psychiatric disturbances, and loss of cognition^20^. It is caused by a CAG repeat expansion in the *HTT* gene^21^ leading to the expression of a mutant huntingtin (HTT) protein that is toxic to the cells and forms protein aggregates^22^. For HD and other neurological diseases, aberrancies in splicing have previously been described^23–26^, suggesting a role for RBPs in neurological pathology. Accordingly, a comprehensive atlas of the RNA interactors responsible for the regulation of post-transcriptional RNA processing events in the brain could lead to novel mechanistic insights to neurological disease.

In the last decade, several mass spectrometry (MS)-based methods allowing for global and unbiased identification of RBPs have been developed^27–29^, with some of these also capable of delineating the specific regions within RBPs directly binding the RNAs^30–32^. However, experimental constraints have until now prevented the application of such methods to the study of RBPs directly in animals or organs.

Here, we describe a streamlined workflow based on the augmentation of our peptide Cross-Linking and Affinity Purification (pCLAP) methodology^32^, which we use to decipher the protein RNA interactome in the mammalian brain. Collectively, we identified 526 RBPs and among these, discovered 86 proteins not previously annotated as RNA-binding nor identified in any global cell culture-based RNA-interactome capture experiment^27–38^, including several synaptic vesicle proteins and metabolic enzymes. Using cross-linking immunoprecipitation (CLIP) analysis^39^ we confirmed the RNA-binding properties of several of these brain-specific RBPs, further demonstrating the utility of brain-pCLAP for studying RBPs in a tissue-specific context. We have further characterized the RBP landscape in brain tissue from the R6/2 HD mouse model^40^ and revealed misregulation of the RNA-binding ability of the alternative splicing factor RBM5^41–43^, despite no change of its expression. Using CLIP analysis, we found that RBM5 binds differentially to several transcripts encoding proteins involved in neurodegeneration or with roles in HD. RNA-seq analysis revealed that these changes correlated with alteration in transcript expression and splicing. Similar changes were also observed in the R6/1 mouse, another model for HD prior to neuronal loss and gliosis^44^. Furthermore, performing loss- and gain-of-function experiments in human neurons, we validated the regulatory role of RBM5 for the splicing changes observed in the HD mice, demonstrating the relevance of our findings in a human context. Finally, co-immunoprecipitation (Co-IP) and proteomic characterization of the protein interactors of RBM5 uncovered a significant overlap between the protein interactors of RBM5 and HTT. Some of these interactions with RBM5 were changed in the HD mouse brain, suggesting a previously uncharacterized link between HTT and the misregulation of RBM5 function in HD. Taken together, this work represents a powerful resource for the identification of tissue specific RBPs and provides the foundation for a better understanding of how post-transcriptional regulation is controlled in tissue and changed during disease.

## Results

### Establishment of the brain-pCLAP methodology

**Fig. 1:**
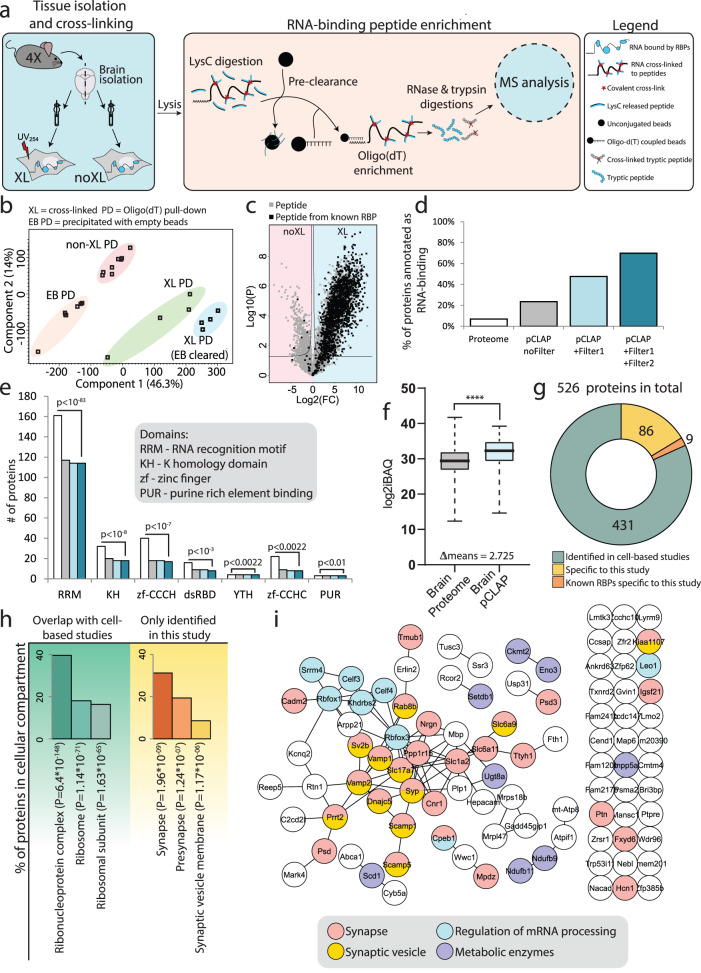
Experimental setup for brain-pCLAP. **a** Experimental workflow of brain-pCLAP: Brain halves from four mice (4X) were homogenized and the right half was cross-linked using UV-light (254 nm). Cells were spun down, and lysates were LysC treated, pre-cleared with empty beads and RNA-peptide complexes were purified using oligo(dT) beads. After elution and trypsin treatment, peptides were analysed using LC-MS/MS. The same experiment was conducted in parallel without the pre-clearance step. **b** PCA plot of MS data from all samples. **c** ‘Filter1’: Volcano plot comparing peptide intensities between cross-linked (XL) and non-cross-linked (noXL) samples (two-tailed *t* test with multiple-testing correction). Each detected peptide is represented by a dot and dots highlighted in black represent peptides from proteins with canonical RBDs. The lines represent thresholds for significant enrichment in the cross-linked (XL) and non-cross-linked samples (noXL). **d** Percentage of proteins previously annotated as RNA-binding in the proteome and proteins inferred from peptides identified by pCLAP before and after the two data filtering steps. **e** Number of proteins with shown canonical RBDs in the proteome and in brain-pCLAP with and without the data filtering steps. The *p* value indicates the enrichment of the fraction of proteins containing each domain in the final brain-pCLAP dataset over the fraction of these protein in the brain proteome^46^ (one-tailed Fischer’s exact test with FDR correction) (colours as in (**d**)). **f** Abundance distribution of total brain proteome and brain-pCLAP. The middle of the box signifies the mean, the box boundaries represent the 25th and 75th percentile and the whiskers reach to the minimum and maximum value of the dataset. *n* = 4 biologically independent animals. *p* value calculated using two-tailed *t* test, ****p < 0.0001. Source data are provided as a Source data file. **g** Donut plot showing the number of proteins identified by brain-pCLAP, which have also been identified as RBPs from cell line based studies and the number of known RBPs among the proteins specific to this study. **h** GO term enrichment for cellular compartment for proteins identified as RBPs specifically by brain-pCLAP or those overlapping with cell line based studies. **i** String network of the 95 proteins identified in this study as RNA binding not identified in previous cell culture-based studies.

To globally identify RBPs and study their dynamics in the brain, we adapted and improved our previously published pCLAP methodology to develop brain-pCLAP. As most other RNA-interactome identification methods, it relies on UV-based cross-linking to capture RNA-protein interactions in vivo^27^. As UV-light does not efficiently reach the inner layers of tissue^45^, gentle dissociation of brain cells prior to UV_254_ irradiation was first performed to ensure efficient cross-linking of the RNA-protein interactions (Supplementary fig. 1a–d). To specifically capture RNA-binding regions, cells were lysed and proteins digested with LysC protease, leaving only the RNA-binding regions cross-linked to RNA. Subsequent pull-down of poly-adenylated RNA using oligo-(dT) beads allowed efficient isolation of these RNA-bound peptides, which were then further digested by trypsin and identified via MS analysis (Fig. 1a). Additionally, a pre-clearance step using unconjugated beads was added to our original workflow, which resulted in lower variance and tighter clustering of replicates (Fig. 1b and Supplementary Fig. 2a–c) and an overall three-fold increase in high-confidence identification of RNA-binding peptides (Supplementary Fig. 2d, e). Altogether, these improvements allowed us to perform pCLAP from the brain (brain-pCLAP) using as little as a single mouse brain for each independent replicate (4 in total) with one hemisphere exposed to UV-cross-linking while the other non-cross-linked hemisphere served as a control.

Peptides significantly enriched in the cross-linked brain samples compared to the non-cross-linked counterparts were first identified as previously described^32^ (referred to as ‘Filter 1’; Fig. 1c). Among these, a strong enrichment of peptides belonging to proteins with canonical RNA-binding domains (RBDs) was observed, confirming that our pull down worked efficiently on brain tissue (Fig. 1c, in black). For additional stringency, a second filtering step (‘Filter 2’) was introduced, for which the ratio of peptides enriched in the cross-linked compared to non-cross-linked samples for each protein passing filter 1 was quantified (see Supplementary Fig. 3 for details) and only proteins with a fraction above 60% were retained. The validity of this approach was supported by the observation that proteins containing canonical RBDs generally had a higher fraction of peptides enriched in the cross-linked samples (Supplementary Fig. 2f, in red), whereas this was not the case for many proteins that are highly abundant in the brain proteome (Supplementary Fig. 2g, in grey).

Among the resulting pool of candidate RBPs, seventy percent have previously been annotated as RNA-binding (Fig. 1d) and constituted a high fraction of all proteins with known canonical RBDs identified in the brain proteome^46^ (Fig. 1e). Together, these results demonstrated the high specificity of brain-pCLAP. Moreover, the stringency of our filtering criteria did not come at the cost of sensitivity, as it excluded a large pool of proteins enriched for very general processes, whilst only removing 3% of proteins containing canonical RBDs (Fig. 1e and Supplementary fig. 2h). To further assess the sensitivity of our methodology, we next compared the sequencing depth of brain-pCLAP to cell culture based RNA interactome studies^28–32,46,47^. This demonstrated that despite the stringency of our workflow, similar depth of sequencing was achieved (Fig. 1f and Supplementary Fig. 2i). Altogether, our results demonstrated that brain-pCLAP enables the identification of the brain RNA-interactome with high specificity and sensitivity.

### The brain RNA-interactome contains candidate RBPs associated with neuronal functions

In total, we confidently identified 526 proteins binding RNAs in the mouse brain. Among these, 431 have previously been identified across 14 different mass spectrometry based RNA-interactome studies from mouse and human cell lines (Fig. 1g and Supplementary Data 1)^27–38^. As expected, gene ontology (GO) term analysis of these proteins showed strong enrichment for ribonucleoprotein complexes (Fig. 1h). The 95 other proteins were enriched for synaptic proteins (Fig. 1h) and also included nine known brain specific RBPs, including RBFOX1, RBFOX3 and SRRM4, with known functions in neuronal development^48–50^. The 86 brain-specific candidates not previously described as RBPs (Fig. 1g) included eight metabolic enzymes and twelve synaptic vesicle proteins (SVPs) (Fig. 1i), nine of which are membrane bound^48^. These observations support the mounting evidence that proteins such as metabolic enzymes can bind RNA, despite this not being their main described function (so called ‘moonlighting’ RBPs)^51^. Our data further suggest that membrane bound SVPs could similarly have ‘moonlighting’ functions as RBPs (Fig. 1i and Supplementary Data 1).

**Fig. 2:**
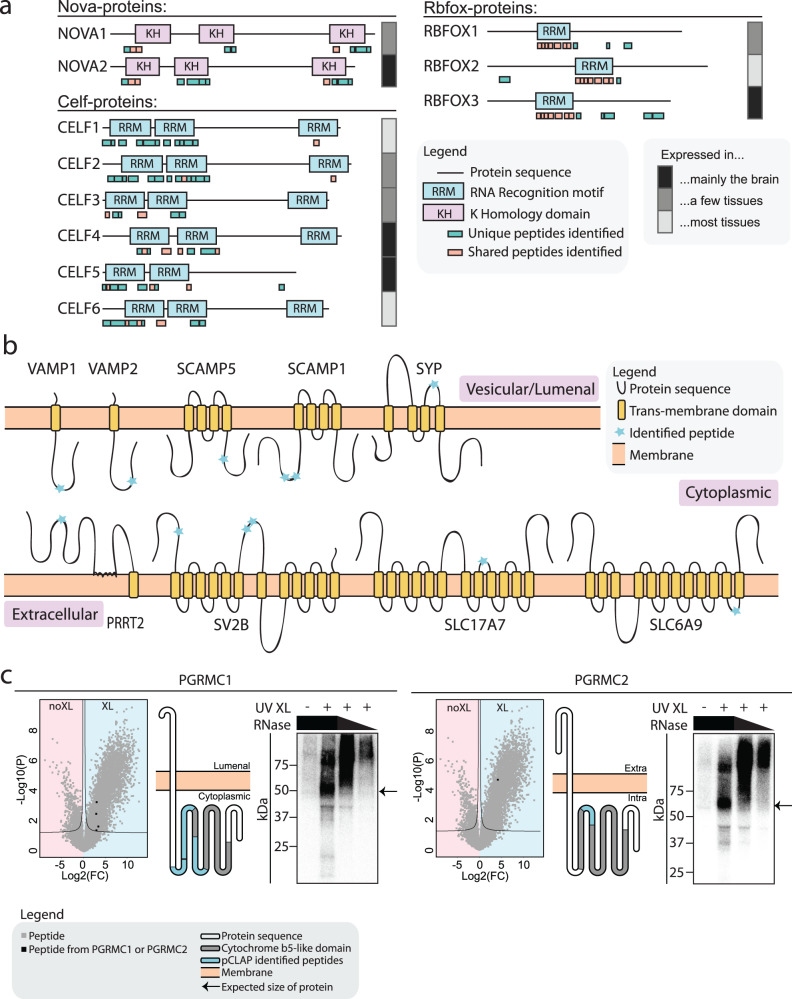
Brain-pCLAP identifies known and novel RBPs. **a** Locations of RNA-binding peptides identified by brain-pCLAP mapped to linear depictions of RBPs. Tissue expression data taken from brain atlas^48^. **b** Schematic of membrane bound synaptic vesicle proteins identified as RNA-binding by brain-pCLAP, showing their trans-membrane domains as well as the locations of peptides identified by brain-pCLAP as RNA-binding regions (blue stars). **c** Data demonstrating RNA-binding ability of PGRMC1 and PGRMC2. Left: Volcano plots (as in Fig. 1c) with peptides from PGRMC1 or PGRMC2 marked in black. Middle: Schematic depiction of both proteins showing their trans-membrane and cytochrome b5-like domains and where the peptides identified by brain-pCLAP are located. Right: CLIP autoradiography for GFP-tagged PGRMC1 and PGRMC2, with visualization of the isotope labelled protein-RNA complex. The left lane is a no cross-link control, followed by the cross-linked samples from highest to lowest concentration of RNase (dilutions 1:10, 1:100 or 1:1000). The arrow points to the expected size of the protein. Each lane of each CLIP experiment represents a replicate of independently grown cells.

We then took advantage of the ability of pCLAP to pinpoint the regions of RBPs that interact with RNA^32^. To demonstrate this ability for brain-pCLAP, all the identified peptides were mapped onto all protein domains reported in the pfam database^52^. From this, we observed a strong enrichment for known RBDs (Supplementary Fig. 4a, b)^46^, with 55% of the peptides mapping to canonical RBDs, and 5% to non-canonical RBDs (Supplementary Fig. 4c, d and Supplementary Data 1), confirming further the translatability of our method to the study of brain tissue. The ability to pinpoint which protein regions are involved in RNA interaction enables brain-pCLAP to further delineate which RBDs are active in the brain. To exemplify this, we first focused on the known brain-specific RBPs from the NOVA, CELF and RBFOX protein families. While brain-pCLAP identified peptides from all the KH and RRM domains across NOVA, CELF1 and CELF2 proteins, no peptides were identified from the third RRM of CELF3, CELF4 and CELF6 (Fig. 2a), although peptides spanning these RRM domains are expressed in the brain proteome^53^. Our brain-pCLAP data therefore suggests that CELF3, CELF4 and CELF6 do not use their third RRM domain for RNA-binding in the brain (Fig. 2a). While we cannot fully exclude that these peptides were not identified for technical reasons, this conclusion is supported by previous studies showing that deletion of the third RRM of these CELF proteins has no effect on their function, whereas deletion of the first two RRMs individually disrupts their splicing activity and/or RNA-binding ability^54,55^. In contrast, both the N-terminal and C-terminal parts of CELF2 can independently act as RNA-binders and splicing factors^54^. Conversely, we identified peptides for RBFOX1 and RBFOX3 in a region not overlapping with known RBDs (Fig. 2a), but known to be able to exert splicing repression function in the case of RBFOX1^56^.

We further used our data to explore which regions of the brain-specific RBP candidates were identified as interacting with RNA. For example, the nine membrane-bound SVPs not previously described as having RNA-binding capabilities were found to primarily bind RNA on their cytoplasmic side (Fig. 2b)^57^. More specifically, based on our data, VAMP1 and VAMP2 were found to both interact with RNA via their v-SNARE coiled-coil domains, which is generally associated with membrane fusion^58^. In conclusion, while revealing the RNA-binding ability of these synaptic proteins, our results also pinpoint their putative RNA-related function to their non-trans-membrane regions.

### CLIP validates RNA-binding ability of candidate RBPs

Cross-linking and immunoprecipitation (CLIP) analysis^39^ was then employed to confirm the RNA-binding ability of several candidate RBPs (Fig. 2c and Supplementary Fig. 4e). Briefly, selected candidates were fused to GFP and expressed in cells. RNA-protein complexes were UV cross-linked and immunoprecipitated with an anti-GFP antibody. The co-immunoprecipitated RNA was radio-labelled with ^32^P and the labelled RBP-RNA complex run and visualized on SDS-PAGE. The RNA content of the complex was confirmed by testing its sensitivity to RNase treatment, using increasing concentrations of enzyme (Fig. 2c and Supplementary Fig. 4f, g). We first confirmed the RNA-binding ability of PGRMC1 and PGRMC2, two structurally similar membrane-bound proteins with a haem-containing cytochrome b5-like domain^59,60^. Our pCLAP data identified this domain as the RNA binder (Fig. 2c), while it is known to be necessary for many of the functions and dimerization of the two proteins^61–64^. For both proteins, an RNase sensitive RNP complex could be detected, collapsing at the expected protein size at the highest RNase concentration used. No RNP complex was observed without cross-linking or for the same experiment done with GFP only (Fig. 2c and Supplementary Fig. 4h). Similar results were observed for other candidates, including the iron-storage protein FTH1, Zinc-finger protein ZCCHC10, synaptic vesicle protein SV2A, as well as membrane bound proteins RTN1 and PRAF2 (Supplementary Fig. 4h)^48,65,66^ and two additional membrane bound SVPs; SYP and VAMP1 (Supplementary Fig. 4i). Collectively, these data further confirmed the ability of the brain-pCLAP methodology to identify RBPs.

### Exploration of the brain tissue of the HD mouse model R6/2 by brain-pCLAP reveals changes in the RNA-binding ability of RBM5

To demonstrate the utility of brain-pCLAP in a disease context, we next applied our workflow to the HD mouse model, R6/2, since perturbations in splicing have been reported in the brains of deceased HD patients and in this representative mouse model^23–26^. We investigated if these defects may derive from aberrant RBP activity by performing brain-pCLAP on four R6/2 HD brains and four wild-type (WT) brains from 12 weeks old sibling mice and comparing the data. As for the previous experiment, we first confirmed that RBPs from both WT and HD samples were identified with high reproducibility, specificity, and sensitivity across all replicates (Supplementary Fig. 5a–c and Supplementary Data 2).

**Fig. 3:**
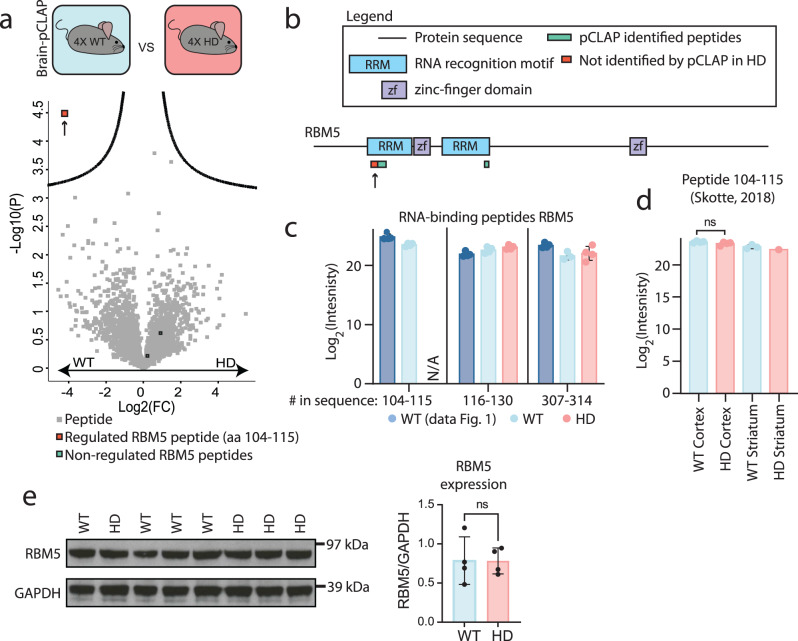
Comparison of active RBPs in a HD and WT mouse brain samples. **a** Volcano plot comparing peptides identified by brain-pCLAP in WT or HD mouse brain samples (two-tailed t-test with multiple-testing correction). **b** Linear representation of RBM5, its domains and brain-pCLAP identified peptides, with the regulated peptide highlighted by an arrow. **c** Average Log2 intensities of RBM5 peptides from all brain-pCLAP experiments in this study. **d** Average Log2 expression intensity of RBM5 peptide spanning amino acids 104–115 in the brain proteome^53^. **e** Western blot of RBM5 and loading control GAPDH from WT and HD mouse brain tissues (left) and quantification of the RBM5 signal normalized to the GAPDH signal (right). For all dot plots, *n* = 4 biologically independent animals. A two-sided *t* test was used, data are presented as mean values +/− SD. Source data are provided as a Source data file.

From the quantitative comparison of the RNA-binding events between the R6/2 HD model and WT mouse brain samples, we identified a single peptide to be significantly affected in its RNA-binding capability in the HD mice (Fig. 3a and Supplementary Data 2). This affected peptide belonged to the alternative splicing factor RBM5^41–43^, a known RBP which contains two RRM domains and two ZF-domains^42^. In total, brain-pCLAP identified three RNA-binding peptides from RBM5 in the WT mouse brain tissue, which mapped to the two RRM domains (Fig. 3b). This is in accordance with previous findings that deletion of the two RRM domains is sufficient to abolish the RNA-binding ability of RBM5^67^. However, only one peptide (spanning the amino acid sequence 104-115) was depleted in the brain-pCLAP samples from the R6/2 compared to the WT brain tissue, suggesting a difference in the RNA-binding ability of this particular RRM in the HD mouse model (Fig. 3a, b). In fact, the affected peptide was not detected in any of the analysed R6/2 samples, whereas it was consistently identified across all eight WT samples analysed throughout this study (Fig. 3c). However, neither the expression of this peptide nor the overall expression of RBM5 in the proteome was changed in the HD mouse brain tissue (Fig. 3d, e, Supplementary Fig. 5d, and Source data)^53,68^. Therefore, the observed change in the RNA-binding ability of RBM5 could not be explained by differential expression of a unique RBM5 isoform or general changes in RBM5 expression in the HD mouse brain tissue. These data imply that the RNA-binding ability of the initial RRM domain of RBM5 undergoes specific alterations in the HD mouse brain, though we cannot discount the possibility that other mechanisms may play a role in this process.

### Enrichment of neurodegeneration and HD pathways among RNAs differentially bound by RBM5 in R6/2 mouse brain tissue

**Fig. 4:**
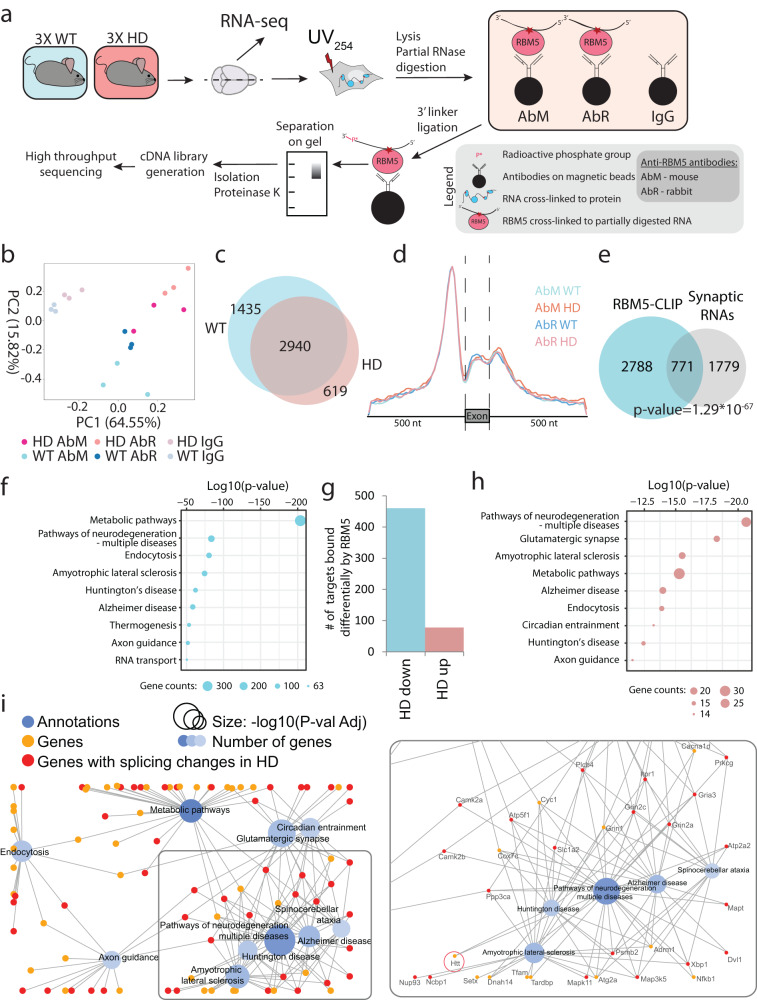
RBM5-CLIP analysis from WT and HD mouse brain tissues. **a** Experimental setup for CLIP analysis from WT and HD brain tissues. Briefly, HD and WT mouse brain tissues were treated with UV-light as for pCLAP. After lysis and partial RNase treatment, RBM5-RNA complexes were isolated using magnetic beads conjugated with either mouse (AbM) or rabbit (AbR) anti-RBM5 antibody and visualised on polyacrilamyde gel. Beads conjugated with unspecific IgG were used as background control. RNA targets bound to RBM5 were then purified and processed by high-throughput sequencing for identification and subsequent analyses. *n* = 3 independent animals for each sample type. RNA-Seq was subsequently performed from the same samples. **b** PCA plot showing RBM5-CLIP data with either antibody and the IgG controls. **c** Number and overlap of RBM5 RNA-targets identified from WT and HD mouse brain tissue (showing targets identified with both antibodies). **d** Metagene distribution of normalized reads from the RBM5-CLIP samples over exons and flanking intronic regions for all sample groups. **e** Overlap of RBM5 RNA targets identified by CLIP and RNAs localizing to the synapse^70^ (*p* value calculated by one-tailed Fischer exact test). **f** GO term enrichment analysis for KEGG pathways on RBM5 RNA targets identified by CLIP. **g** Number of genes encoding transcripts that are bound differentially by RBM5 in HD. **h** GO term enrichment analysis for KEGG pathways for the transcripts bound differentially by RBM5 in HD (*p* values in **f**, **h** were calculated using one-sided Fischer’s exact test). **i** Network of the 10 most significantly enriched GO KEGG pathways among genes encoding transcripts bound differentially by RBM5 in HD, where the GO terms are presented as blue nodes and genes falling under these terms are shown as yellow and red nodes connected with an edge to the nodes. Neurodegeneration relevant terms enlarged on the right.

Next, we wanted to further investigate the RNA-binding capabilities of RBM5 in the HD model. To this end, we established high-throughput sequencing (HITS)-CLIP^39^ on endogenous RBM5 using two different antibodies (AbM and AbR), and applied it to WT and HD mouse brain tissues (Fig. 4a and Supplementary Fig. 6a, b). Principal component analysis (PCA) showed that the samples grouped closer by genotype rather than by antibody-type supporting the implementation of a successful CLIP experiment (Fig. 4b). For stringency, only read clusters identified in both antibody experiments (AbM and AbR) and showing at least two-fold enrichment over the IgG controls were considered genuine RBM5 binding sites (Supplementary Fig. 6c). From this, we identified 4375 and 3559 transcripts as RBM5 targets from WT and HD mouse brain samples respectively (Fig. 4c, Supplementary Fig. 6c, and Supplementary Data 3). For both genotypes, we found that ~60% of the RBM5-binding sites located to introns, with a strong enrichment less than 100 nt upstream of the 3’ splicing site (ss) in relation to intron–exon junctions (Fig. 4d). This is in agreement with reports showing that RBM5 regulates the utilization of weak 3’ss^41,67,69^. GO-term analysis of RBM5 targets showed enrichment of pathways for neurodegeneration, including HD, while a significant number of RBM5 targets have been shown to localize to the synapses for localized translation (Fig. 4e, f)^70,71^. In agreement with *RBM5* being a pro-apoptotic tumour suppressor gene^67,72,73^, caspase and cyclin transcripts were also bound by RBM5 (Supplementary Fig. 6d). Comparing the targets for the two genotypes, we identified 538 transcripts differentially bound by RBM5 in the R6/2 mouse brain tissue (Fig. 4g). These differentially bound transcripts were further enriched for pathways of neurodegeneration and HD (Fig. 4h). Network visualization of the ten most significantly enriched GO terms showed that many of these transcripts have roles in several interconnected neurodegenerative diseases (Fig. 4i).

### Expression and splicing of RBM5 targets are altered in the R6/2 mouse brain

**Fig. 5:**
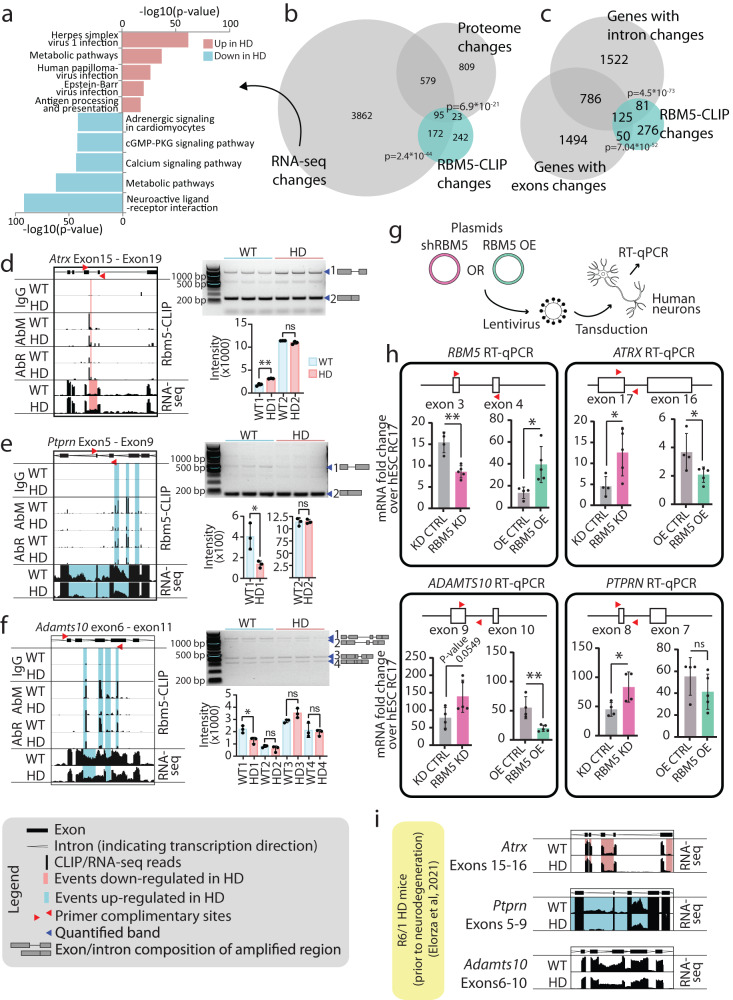
Changes in transcriptome and proteome in HD coincide with changes in RBM5 RNA-binding. **a** GO terms enrichment analysis for KEGG pathways for transcripts regulated in HD (*p* value: Fischer’s exact test with FDR correction). **b** Overlap of significant changes in HD mouse brains — RBM5-binding changes detected by CLIP analysis, transcriptome expression changes and proteome expression changes^53^ (*p* values: one-tailed Fischer’s exact test corrected for multiple testing). **c** Overlap of transcripts bound differently by RBM5 in HD and transcripts with intron or exon inclusion changes in HD (*p* values: one-tailed Fischer’s exact test corrected for multiple testing). **d**–**f** Data demonstrating RBM5 binding changes and splicing changes for *Atrx*, *Ptprn* and *Adamts10*. Left: Transcript regions with significant changes in RBM5-CLIP data and RNA-seq data, with the gene model shown on top. Top six lanes show RBM5-CLIP data: top two lanes show IgG controls, followed by RBM5-CLIP data acquired with either antibody (AbM and AbR) for both genotypes. Bottom two lanes show RNA-seq data for both genotypes. Significantly regulated RBM5-binding events or intron/exon inclusion in HD are highlighted. Top right: Gels showing PCR amplification products (location of primers used for PCR indicated with red arrowheads on gene model shown on the left) and the region of the corresponding isoform amplified (indicated on the right of the gel). Bottom right: Average intensity of quantified gel bands. *n* = 3 independent animals for HD and WT samples. **g** Schematic representation of *RBM5* KD and OE using lentivirus transduction with *RBM5* KD or OE vectors. **h** Normalized intensities for qPCR on human neurons amplifying an exonic region for *RBM5* and intronic region for *ATRX*, *ADAMTS10* and *PTRPN* in *RBM5* KD and OE neurons. Primer locations are indicated with red arrowheads. *n* = 5 biologically independently differentiated and transduced replicates. **i** Analysis of previously published RNA-seq data from slower progressing HD R6/1 mouse model^44^ (prior to neuronal loss) for the same regions presented for *Atrx*, *Adamts10* and *Ptprn* in **d**–**f** above. For all dot plots, data are presented as mean values +/− SD, *p* values: two-tailed *t* test, *p < 0.05, **p < 0.01, ns = not significant (source data in Source data file).

We next investigated if the differential binding of RBM5 was associated with a change of expression of its targets by performing RNA-seq transcriptome analysis on brain tissue from the same WT and HD mice used for the CLIP analysis. As expected, PCA analysis showed grouping of the samples by genotype (Supplementary Fig. 6e), with more than 3000 transcripts differentially expressed in R6/2 mouse brain tissue (Supplementary Fig. 6f and Supplementary Data 4). GO-term analysis of these transcripts showed enrichment of genes involved in metabolic pathways, synaptic signalling pathways and viral and immune responses (Fig. 5a), which is in agreement with previous reports^53,74,75^. Interestingly though, and contrary to RBM5-CLIP, we did not observe specific enrichment for neurodegeneration pathways genes, suggesting further a specific association of RBM5 with neurodegeneration relevant transcripts. Comparison between our RNA-seq data, our RBM5-CLIP changes and published brain proteome data from the same R6/2 HD strain^53^ showed a statistically significant overlap. For 55% (290 of the 532) of the transcripts differentially bound by RBM5 in the R6/2 mouse brain, we also observed changes in expression levels for the corresponding transcripts or proteins (Fig. 5b and Supplementary Fig. 6g).

Our transcriptome data furthermore revealed that ~3500 RNAs were differentially spliced, with significant changes in the inclusion of specific exons or introns (Supplementary Fig. 6h and Supplementary Data 4). Comparison between the differentially spliced transcripts and the transcripts bound differentially by RBM5 in R6/2 mouse brains revealed a significant overlap between the two datasets (Fig. 5c), including many neurodegeneration-related genes (Fig. 4i), suggesting a role for RBM5 in regulating the splicing of these genes.

To confirm that our observations indeed represent changes in alternative splicing, we chose three transcripts (*Atrx, Ptprn* and *Adamts10*) differentially bound by RBM5 and differentially spliced in the diseased brain for further investigation (Fig. 5d–f; left side). In accordance with the RNA-seq data, we also observed splicing changes of specific introns for all three candidates via RT-PCR analysis (Fig. 5d–f; right side), confirming that the differential splicing correlated with RBM5 binding changes in the R6/2 mouse brain. Altogether, our results demonstrate that RBM5 binds specific RNA targets in the brain, including neurodegenerative-related transcripts, and that the binding to these targets and their splicing are affected in a mouse model of HD.

Finally, in agreement with proteomics and western blot data, we did not see any changes in the expression or splicing of RBM5 itself (Supplementary Data 5), confirming further that the change in RBM5 function in the R6/2 mouse brains is uniquely regulated at the level of its RNA-binding ability.

### Splicing changes observed in R6/2 mouse model were demonstrated to be regulated by RBM5 in human neurons, and detected in another HD mouse model as well as in HD patient samples

The experiments in R6/2 mice demonstrated that RBM5 binding changes significantly overlapped with splicing changes of its target RNA. To ascertain the causal link between these events, we tested if the splicing of RBM5 targets was affected upon knock-down (KD) or over-expression (OE) of *RBM5*.

To also further extend the analysis to human cells, we differentiated human embryonic stem cells (hESCs) towards lateral ganglionic eminence (LGE) fate, as the LGE contains the most affected neuronal subtypes in HD^76,77^. *RBM5* was then KD or OE using lentiviral transduction (Fig. 5g). Five independent differentiations and transductions were performed. *RBM5* KD and OE were validated by RT-qPCR (Fig. 5h and Supplementary Fig. 7a). Moreover, proper differentiation and maturation of the neurons in all conditions were validated by confirming the expression of the expected neuronal markers (Supplementary Fig. 7a). We then analysed splicing of the human *ATRX, PTRPN* and *ADAMTS10* transcript introns corresponding to the same introns affected in their orthologs in R6/2 HD mice. Doing so, we observed an increase in intron retention upon *RBM5* KD and a decrease upon *RBM5* OE (Fig. 5h). Importantly, these changes occurred without any modification of expression of the exonic regions adjacent to the intron or further downstream (Supplementary Fig. 7b), confirming a change specifically in splicing and not of the general expression levels of the transcripts. Collectively, these data confirmed that RBM5 regulates the alternative splicing changes observed in the R6/2 mouse brain and furthermore, we demonstrate that this regulation is conserved in human neurons.

To confirm our findings in another HD model and to assess whether the alternative splicing changes regulated by RBM5 are an early or late attribute of HD, we next analysed published RNA-seq data from the R6/1 mouse model^44^, mimicking a milder HD phenotype, at a stage before neuronal loss and gliosis. Doing so, we observed the same intron retention events for *Atrx* and *Ptprn* as observed for the R6/2 mice and in human neurons (Fig. 5i), demonstrating that these RBM5 driven alternative splicing events are misregulated already in earlier stages of HD. We additionally observed a significant overlap between RBM5 binding changes in the R6/2 mouse (our study) and differential intron and exon inclusion events in the R6/1 mouse model^44^ (Supplementary Fig. 6i). This supports that RBM5 misregulation and the resulting alternative splicing changes are early attributes of HD occurring prior to neurodegeneration, rather than late downstream consequences of the disease. Finally, we analysed published RNA-seq data from HD patient samples^44^ and observed a significant overlap between differential intron and exon inclusion events in these with RBM5 binding changes in R6/2 mice (Supplementary Fig. 6j, k).

### Interactions between RBM5, RNA processing factors and Huntingtin interactors are perturbed in the R6/2 mouse brain

**Fig. 6:**
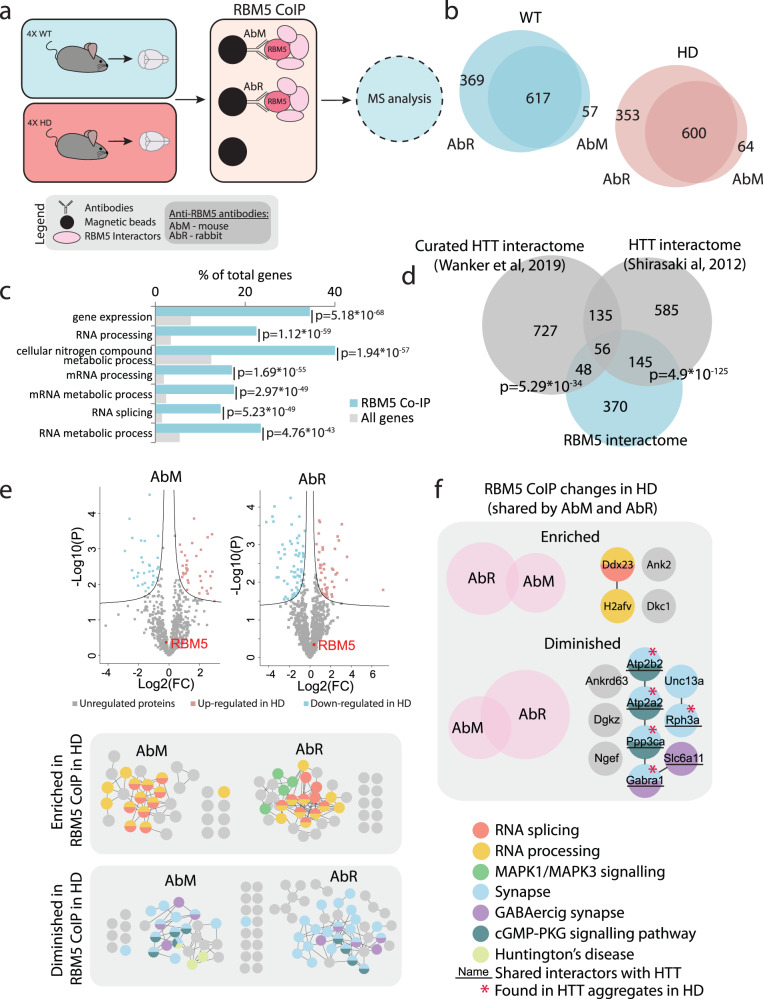
RBM5 interactome of the brain in WT and HD. **a** Experimental setup for RBM5 AP-MS from brain tissue. HD and WT mouse brain tissues were lysed in non-denaturing conditions and RBM5 together with its interacting proteins were isolated using magnetic beads conjugated with either mouse (AbM) or rabbit (AbR) anti-RBM5 antibody. Empty beads were used as background control. The interactors of RBM5 were identified using mass-spectrometry. **b** Number of interactors identified for RBM5 with both antibodies from WT and HD brain tissue (only the proteins identified with both antibodies were considered true interactors). **c** GO term enrichment analysis for biological processes for the 619 RBM5 protein interactors (WT and HD combined). **d** Overlap between the proteins identified in this study as RBM5 interactors (WT and HD combined) in the mouse brain and HTT interactomes from previous publications^79, 80^ (one-tailed Fischer’s exact test). **e** Comparison between the WT and HD RBM5 protein interactome separately for AbM and AbR. Top: Volcano plot comparing the RBM5-interactomes in WT and HD. Bottom: Proteins interacting with RBM5 differently in HD shown in networks (two-sided *t* test with multiple-testing correction). **f** Venn diagrams showing the overlap of RBM5 protein interactions differentially regulated in HD identified with both antibodies and network representation of the overlapping proteins.

Since RBM5 expression was not changed in the HD mouse brain, we wanted to explore what may be causing the specific misregulation of its RNA-binding ability. RNPs are composed of a defined combination of proteins fine-tuning the activity of each other^3^ and the polyglutamine expansion in HTT introduces perturbations in the proteome^22^. We therefore reasoned that the misregulation of RBM5 in controlling splicing in the R6/2 brain may be caused by an altered interaction with its protein partners. To test this hypothesis, we identified the RBM5 protein-interactome via affinity-purification mass spectrometry (AP-MS) from WT and HD mouse brain samples using the same two anti-RBM5 antibodies (AbM and AbR) used for CLIP (Fig. 6a).

Overall, we obtained excellent reproducibility between replicates and samples clustered based on genotype on a PCA plot (Supplementary Fig. 8a, b). After filtering out unspecific binders and considering only interactors significantly enriched with both antibodies (Fig. 6b and Supplementary Fig. 8c, d), we identified a total of 617 RBM5 protein interactors in the WT brain and 600 in the HD brain, with high overlap between the two antibodies, providing a total of 619 protein interactors (Fig. 6b and Supplementary Data 6). In agreement with the literature, RBM5 interactors were highly enriched in RNA processing and splicing factors (Fig. 6c), including U2AF2 (U2AF65), a protein known to interact with RBM5 to co-regulate several splice events^43,69,78^. Interestingly, a significant number (40%) of the identified RBM5 interactors also constitute known HTT interactors (Fig. 6d)^79,80^, with the shared interactors between RBM5 and HTT highly enriched for proteins involved in mRNA metabolic processes, neuron specific processes and Huntington’s disease (Supplementary Fig. 8e). This was specific to RBM5, as a much lower overlap was observed between proteins interacting with HTT and the interactomes of three RBPs unrelated to this study (Supplementary Fig. 8f)^81–83^.

Finally, we uncovered 14 proteins that exhibit altered interaction with RBM5 in the HD mouse brain, including several RNA processing and splicing factors as well as synaptic signalling factors (Fig. 6e, f and Supplementary Data 6). One of these, DDX23 is a splicing factor necessary for the transition of the spliceosome to its active form, whereas by contrast, RBM5 has been reported to block this transition^42,84^. Among the other regulated RBM5 interactors, six of the proteins that interact less with RBM5 in the HD brain constitute known interactors of HTT, with five of these also reported to localize to HTT aggregates^22,68,79,80^ (Fig. 6f). Taken together, our data therefore demonstrate that the protein-protein interaction landscape of RBM5 is disturbed in the HD mouse brain, which could be a cause for RBM5 misregulation. It further suggests that this perturbation could be caused by a change of availability with interactors known to be sequestrated into HD-related aggregates.

## Discussion

RBPs can have brain specific functions and their malfunctioning is a known contributor to neurological disorders^6,16^. To understand the range of RBP functions and their contribution to disease in its whole complexity, it is critical to study RBPs directly in tissues and organs. In this study, we report the development of brain-pCLAP that allows studying the RNA-binding ability of proteins globally in tissue. Brain-pCLAP provides two dimensions of information. Firstly, it allows global identification of RBPs and their RNA-binding regions in the brain. This offers a basis for further studies and broader understanding of proteins regulating post-transcriptional processes in this organ. Here, we demonstrated the RNA-binding ability of several brain-specific proteins not previously characterized as RNA-binding, including several metabolic enzymes and synaptic vesicle proteins, providing further evidence in support of proteins ‘moonlighting’ as RBPs. Secondly, brain-pCLAP provides information on disease related changes across RBPs and individual RNA-binding regions. This can be used to unbiasedly identify RBPs that have compromised functions in disorders affecting the brain. We demonstrated this by applying brain-pCLAP to the R6/2 HD mouse model and found that the RNA-binding ability of the alternative splicing factor RBM5 is altered. Furthermore, the ability of brain-pCLAP to identify specific RNA-binding regions of RBPs can reveal subtle regulatory changes, not associated with change in expression or deleterious mutations, which would therefore evade classical proteomics or sequencing approaches. In line with this, while RBM5 expression was unchanged, as shown by us and others^46,53^, we observed that the RNA-binding properties of the first RRM of RBM5 were specifically altered in HD mice.

RBM5 is a ubiquitously expressed RBP known to regulate mRNA and protein levels, as well as alternative splicing of genes involved in regulation of apoptosis, cell cycle, cytoskeleton, and drug metabolism^43,69,73,85–87^. RBM5 can both stimulate and suppress splicing at specific splice sites via different mechanisms^41,42,67,69,72,88^. Notably, these opposite mechanisms involve different protein-protein and RNA-protein interactions, suggesting a complex mechanism of regulation for the activity of RBM5. One characteristic of RBM5 that could allow for highly specific modes of action is that its RBDs seem to have distinct regulatory roles, with mutations in either RRM domain shown to differently affect RBM5 function and specificity^69^. Furthermore, the second RRM domain of RBM5 has been shown to adapt different conformations leading to different target specificities^89^, suggesting a mechanism through which the individual RRMs could regulate the function of RBM5. In support of the separate functional roles of the RRM domains of RBM5, we find that the RNA-binding properties of the first RRM are altered in HD mice and consequently report the identification of ~500 target transcripts bound differentially by RBM5 in R6/2 mouse brain tissue. These targets are significantly enriched for transcripts involved in neurodegenerative diseases, including HD, and overlap with changes in transcript and protein expression levels and splicing changes. This suggests a mechanism where the misregulation of the function of the first RRM of RBM5 in the HD mouse brain may lead to aberrant expression and splicing of several of its HD relevant targets (Supplementary Fig. 9), which could contribute to the aetiology of this disease. Although the consequences of *RBM5* misregulation have been demonstrated to affect very different biological contexts, including tumour proliferation, fertility or traumatic brain injury, the proposed underlying mechanism for all of them is the same: misregulation of apoptotic genes leading to apoptosis^43,90^. Here, our data suggest that *RBM5* may have a role in directly regulating the expression and splicing of transcripts relevant to neurodegenerative diseases, supporting a role for *RBM5* in neurodegeneration via other pathways than apoptosis.

**Fig. 7:**
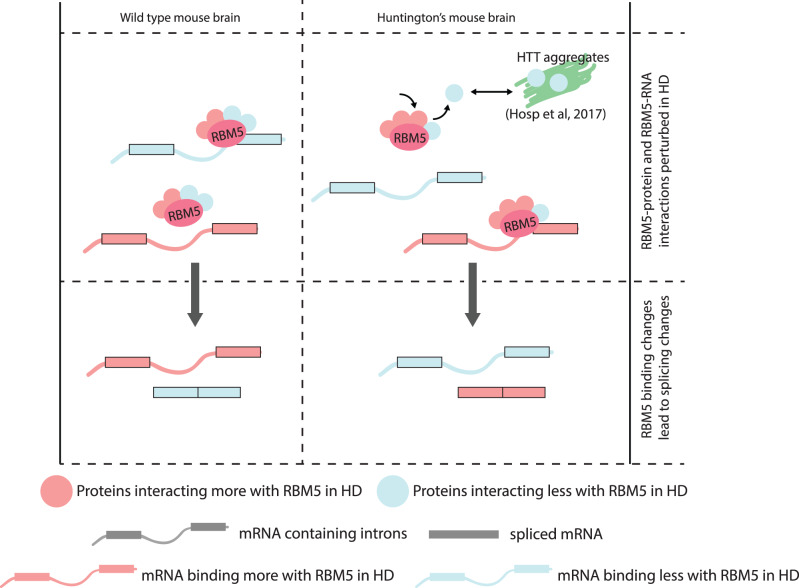
Working model. The protein–protein interaction landscape involving RBM5 is perturbed in the HD mouse brain, where some proteins interact more (light pink) while others interact less (light blue) with RBM5. Some of the proteins interacting less with RBM5 are also components of HTT aggregates, which could explain the change to the RBM5 protein interactome in HD. Meanwhile RBM5 binds less to some transcripts (light blue) and more to others (light pink) in the HD mouse brain. The change in RBM5 binding to these transcripts then leads to﻿﻿ changes in their splicing.

Furthermore, we have demonstrated that the RBM5 protein-protein interaction landscape is perturbed in the HD mouse model. Considering firstly that no *RBM5* expression, splicing or isoform changes were observed in the HD mouse brain in this or previous studies in R6/2 mice^46,53^ and secondly that RBPs function in concert with one another^3^, we propose that the changes in the interaction of RBM5 with other proteins could lead to the misregulation of the RNA-binding ability of RBM5 observed in R6/2 mouse brains. Intriguingly, among the 14 proteins interacting differentially with RBM5 in the disease context, we observe decreased interaction with six proteins that have previously been identified to interact with HTT, five of which are found in HTT aggregates^68,79,80^. The molecular mechanisms of how the reduced availability of several RBM5 protein interactors sequestered into HTT aggregates affects RBM5 function should be interesting to investigate in future studies. Conversely, we observe increased interaction between RBM5 and DDX23. The latter is required for the transition of the spliceosome from its inactive confirmation to its active confirmation^84^, whereas RBM5 has been shown to prevent this transition^42,88^. This suggests regulatory interplay between the two proteins, which could be perturbed in HD and could contribute to the splicing changes observed. Altogether, our multi-omics study allows us to suggest a working model where the changes of interaction between RBM5 and its protein partners would lead to the misregulation of its RNA-binding ability, leading to aberrant splicing of its targets (Fig. 7). This includes many neurodegenerative- and HD-related RNAs, whose dysregulation could potentially affect the overall expression and function of several genes contributing directly to HD pathology (Supplementary Fig. 9).

Finally, we demonstrated that many transcripts bound differentially by RBM5 in the brain of R6/2 mice undergo alternative splicing changes also in R6/1 mice, mimicking HD before the signs of neurodegeneration^44^, and in HD patients^44^. This suggests that RBM5 misregulation and the resulting alternative splicing changes are an attribute of early HD development and not a secondary consequence of the disease. Furthermore, we showed that the alternative splicing events that are altered in the HD mouse brains are regulated by RBM5 in human neurons differentiated from stem cells, demonstrating the potential implication of our findings to the human brain context.

While our data all point to a change in the RNA-binding ability of RBM5 in HD, we cannot exclude other explanations overlooked by the limitation of our methods and analyses. Additional investigations using human samples will also be needed to further characterize the contribution of RBM5 misregulation to HD in patients. Lastly, as standard practice in the field when this work was initiated, only male mice were studied to reduce variation and to avoid gender specific effects that could not be pinpointed in our current setup. Therefore, and while some of our findings were supported by experiments done in female cell lines or using data from human HD patient from both sexes, we cannot fully exclude that some of our observations are limited to male individuals.

In conclusion, we developed the brain-pCLAP methodology, which allowed establishment of a comprehensive atlas of RBPs in the mammalian brain. This represents a powerful resource for understanding the mechanisms of post-transcriptional regulation and gene expression control in the brain. Our methodology allows the identification of the RBP interactome of metazoan cells under native conditions without the need for expansion in culture, which is an important technological milestone in the field of RNA biology. Finally, we identified and validated RBM5 as an aberrant RNA binder in models of HD, explaining changes observed in alternative splicing for this disease and demonstrating the utility of our approach to study RBP function in neurodegenerative disorders.

## Methods

### Mouse strains

R6/2, transgenic for exon 1 of the human HTT gene^40^, originated from The Jackson Laboratory (Bar Harbor, ME, USA) and were maintained by backcrossing males to CBA/j3B6 females (Taconic, Denmark). The mice were kept under specific pathogen-free conditions at a 12 h light/12 h darkness cycle, at 21 °C (20–24 °C temperature range) and at a humidity of 55 ± 10% in standard polystyrene cages with ad libitum access to standard chow and water. DNA was extracted from ear punches and used for genotyping^91^. The repeat length was between 190 and 200 CAG throughout the experiment. Twelve-week-old male mice were used for all experiments. WT littermates without the HTT transgene were used as controls. Experiments were performed in accordance with the Danish Animal Experiments Inspectorate’s guidelines (permit 2007/561-1345), the Danish Working Environment Authority (permit 20070033239/4), and European Commission Directive 86/609/EEC for animal experiments.

### Isolation of mouse brains, tissue processing and homogenization for brain-pCLAP 

The mice were euthanized by cervical dislocation and the brains were collected on ice. The two brain hemispheres were separated and each cut into four pieces. The pieces from each hemisphere were moved to a Tenbroeck homogenizer on ice, containing 5 mL of ice-cold HBSS without Ca^++^ and Mg^++^ (14175129; Thermo Fischer Scientific) and the brain cells were dissociated from the tissue by three gentle strokes with the homogenizer. The suspension was subsequently passed through a 50 µm nylon filter (04-004-2327; Sysmex America) using a syringe plunger, resulting in a single cell suspension. The homogenizer and filter were then both washed with HBSS (-Mg, -Ca) to collect all cells. The cells from the right hemisphere were UV-cross-linked twice with UV-light at 254 nm 0.175 J/cm^2^ (Dr. Gröbel GmbH), whereas cells from the left hemisphere were used as negative control and not UV-treated. In all other respects, the two halves were treated in the same way. The cell suspension was then spun down at 4 °C at 500 × *g* for 10 min and followed by immediate freezing of the cell pellet on dry ice. Samples were stored at −80 °C.

During this study, we tried nine different approaches for the separation of brain tissue into single-cell suspension. The sample yield for each approach was quantified using the Bradford protein assay and the cross-linking efficiency was assessed by conducting pCLAP analysis as described previously^32^ and comparing peptide intensities in the cross-linked and non-cross-linked samples to each other (Supplementary Fig. 1).

### Tissue lysis for brain-pCLAP

Cells were lysed with lysis buffer containing 6 M GndHCl, 100 mM Tris (pH 8.0), 1 mM EDTA, 5 mM tris(2-carboxyethyl)phosphine (TCEP; C4706, Sigma Aldrich), 10 mM chloroacetamide (CAA; 22790, Sigma Aldrich). Lysis was followed by immediate sonication at 70% amplitude (Sonics) three times for 20 s. Samples were kept on ice between the sonication cycles. Samples were cleared by centrifugation at 14,000 × *g* for 15 min at 4 °C and the supernatant was collected. Protein concentration was measured using the Bradford protein assay and 8–10 mg total protein was used per pull-down. LysC (Wako; protein:LysC ratio = 100:1) was added to each sample and LysC digestion was performed for 4 h at room temperature. Samples were cooled down on ice and diluted to a GndHCl concentration of 2 M with a dilution buffer (100 mM Tris (pH 8.0), 1 mM EDTA, 5 mM TCEp, 10 mM CAA).

### Immunoprecipitation with oligo(dT) for brain-pCLAP

For pre-clearance, half of the lysed brain samples (4 cross-linked and 4 non-cross-linked) were incubated with empty magnetic beads (Custom made, NEB) for 1 h at 4 °C on a rotator. The beads were pelleted on a magnetic rack (DynaMag-2 Magnet, 12321D, Thermo Fisher Scientific). The cleared supernatants were used for oligo(dT) pull-downs. For enrichment of RNA and the cross-linked LysC peptides representing the RNA-binding regions of the proteins in the brain, the samples were incubated with magnetic oligo(dT) beads (S1419S, NEB) at 4 °C on a rotator for 1 h. The beads were pelleted using the magnetic rack. Beads were washed three times with washing buffer (2 M GndHCl, 100 mM Tris (pH8.0), 1 mM EDTA, 5 m MTCEp, 10 mM CAA). After the last wash, the supernatant was removed and the beads were pelleted with a quick spin. Samples were heat eluded at 80 °C in elution buffer (20 mM Tris (pH 8.0), 1 mM EDTA) on a shaker at 400 rpm. NaCl was added to each sample to a concentration of 0.15 M followed by RNase treatment using a mixture of RNase A and T1 (EN0551; Thermo Scientific), the samples were incubated for 1 h at 37 °C on a shaker at 350 rpm. This was followed by addition of trypsin (Sigma; peptide:trypsin ratio = 100:1) to each sample and incubation overnight at 37 °C on a shaker at 350 rpm. Samples were acidified by addition of 10% Trifluoroacetic acid (TFA) to lower the pH of the sample to 2–3, followed by pelleting all undigested proteins by centrifugation at 14,000 × *g* for 15 min at 4 °C. The pellet was discarded and the supernatant was used for stage tipping.

During this study, we also tested brain-pCLAP without the pre-clearance step with empty beads, which is shown for comparison in Fig. 1b and Supplementary Fig. 2a–e (noted as XL PD). We also saved and eluded the peptides captured with the empty beads during the pre-clearance and analysed them by MS, these are shown for comparison in Fig. 1b and Supplementary Fig. 2a–c (noted as EB-PD).

### Clean-up and concentration on SCX and C18 StageTips for brain-pCLAP

Digestet RNA was removed using SCX StageTips. Four disks of SCX material (Empore SPE disks, 66888-U, Sigma Aldrich) were pushed into a p200 pipette tip. The SCX material was activated by individually passing 200 µL of the following buffers in the specified order through the material, using centrifugation (800 × *g*): MeOH, 20% Acetonitrile (ACN, 34967-2.5L, Sigma Aldrich) with 0.1% TFA, SCX-buffer pH 12 (20% ACN, 20 mM boric acid, 20 mM phosphoric acid, 20 mM acetic acid, pH adjusted to 12 with NaOH) and 20% Acetonitrile/ 0.1% TFA. StageTips were not centrifuged to complete dryness at any point. The samples were loaded onto the activated SCX StageTips and the flow-through was discarded. The peptides were eluded with 200 µL SCX-buffer pH 12 and concentrated to 100 µL until the ACN was evaporated using Vacufuge (Eppendorf). C18 StageTips were used to desalt and concentrate the samples as described before^92^. Two disks of C18 material (Empore SPE Discs, 66883-U, Sigma Aldrich) were pushed into a p200 pipette tip. The C18 material was activated by individually passing 100 µL of the following buffers in the specified order through the material, using centrifugation (800 × *g*): MeOH, 80%, ACN with 0.5% Acetic acid, 0.5% of Acetic acid. The concentrated samples were loaded onto the activated C18 StageTips and washed once with 0.5% of Acetic acid. The loaded StageTips were run dry and stored in the fridge. Samples were eluded right before MS using 40 µL of 40% ACN and 0.5 % Acetic acid. The samples were concentrated to below 8 µL and the acetonitrile evaporated in a Vacufuge at 45 °C. 1 µL or more of 5% Acetonitrile and 0.1% TFA (T6508-500ML, Sigma Aldrich) was added to the samples to acidify the sample and bring the final volume to 9 µL.

### MS analysis of brain-pCLAP samples

Each technical replicate was analysed as a single shot on the Q Exactive HF (Thermo Fischer) mass spectrometer coupled to an EASY-nLC 1200 System (Thermo Fischer) with 15 cm long in-house pulled and packed column, filled with ReproSil-Pur 120 C18-AQ, 1.9 µm (r119.aq; Dr. Maisch). Peptides were separated out on a 77-min low pH gradient with a mobile phase of 0.1% Formic acid (1.00264.1000, VWR) and an Acetonitrile concentration gradient ranging from 0% to 80%. The mass spectrometer was operated using the ‘sensitive method’ described previously^93^. More precisely, ions were accumulated to a target value of 1e6 and MS1 spectra (*m*/*z* 300–1750) were acquired using the Orbitrap at a resolution of 70 000 resolution (for *m*/*z* 200). The 12 most intense ions that were multiply charged were isolated with a fixed injection time of 120 ms, fragmented by higher-energy collisional dissociation (HCD) with HCD collision energy 25% and analysed in the orbitrap with a resolution of 35 000 resolution.

### Brain-pCLAP data analysis

Raw mass spectrometry data files were analysed by the MaxQuant^94^ (v1.4.1.1 and v1.5.0.38) software suite using standard settings, supported by the Andromeda search engine^95^. Briefly, data were searched against a concatenated target/decoy^96^ (forward and reverse) version of the UniProtKB (11.07.2016) database encompassing 71,434 protein entries. We used the step-by-step protocol of the MaxQuant (version 1.5.0.38) software suite to generate MS/MS peak lists that were filtered to contain at most six peaks per 100 Da interval prior to the Andromeda database search. The mass tolerance for searches was set to a maximum of 7 ppm for peptide masses and 20 ppm for HCD fragment ion masses. Data were searched with carbamidomethylation as a fixed modification and protein N-terminal acetylation and methionine oxidation as variable modifications. A maximum of two miscleavages were allowed while requiring strict trypsin specificity^97^. All the replicates were analysed together, and match between runs was turned on. Reverse hits and contaminants were removed from the protein and peptide data. The peptide.txt output file was then analysed in Perseus (version 1.5.1.2 and 1.6.1.2.; Max-Planck Institute of Biochemistry, Department of Proteomics and Signal Transduction, Munich, Germany). Pearson correlations between replicates and samples from different sample groups were calculated using column correlations in Perseus. Peptides that had intensities from less than three replicates in one of the sample groups were removed before statistical analysis. Missing values, including intensities for peptides identified in only the cross-linked or non-cross-linked sample, were imputated on the basis of the normal distribution of the intensities in each sample using standard parameters in Perseus. All further analysis was done with the four cross-linked and non-cross-linked replicates from the pre-cleared samples.

The cross-linked samples were compared with the non-cross-linked sample using the two-tailed Student’s *t* test in Perseus, with an FDR of 0.05 and s0 of 0.1, to determine which peptides were significantly enriched in the cross-linked samples. Gene ontology (GO) and InterPro motif enrichment analyses were conducted using GeneCodis^98^ and PANTHER version 11^99^. Peptide localization in proteins was assessed using MaxQuant Viewer and Perseus by mapping sequences onto known Pfam domains as reported in the Pfam database^52^.

Abundance distribution analysis was done by extracting iBAQ values for each protein identified by brain-pCLAP from a deep brain proteome data^46^, which signifies the abundance of the proteins in the brain proteome. The same was done for cell-based RNA-interactome capture studies, where iBAQ values were extracted from a deep HeLa proteome^47^. Differences in protein abundance distributions between brain-pCLAP and the brain proteome and pCLAP and other cell-based RNA-interactome studies compared to the HeLa proteome were calculated using a two-tailed, unpaired *t* test using the program Prism. The mean for the brain proteome was 29.31 and 24.7 for the HeLa proteome. The means of the other samples correspond to either brain proteome or HeLa proteome shown plus the mean difference shown under each box in the figure. *p* Values for comparisons of abundance distribution differences between Brain proteome vs. brain pCLAP and HeLa proteome vs. cell-based RNA-interactome capture experiments were given as <0.0001 by Prism.

For the R6/2 (HD) comparison to WT littermates, all peptides identified as enriched in the cross-linked sample for either WT or HD or both were retained in the dataset and before comparison and subtracted mean normalization was done in Perseus, assuming that the overall intensity of the samples should be similar. The differences were assessed using two-tailed Student’s *t* test in Perseus (version 1.5.1.2)^100^.

### Culturing and transfection of HEK293T cells

Human embryonic kidney cells (HEK293T cells (CRL-3216, female), acquired via the American Type Culture Collection.) cells were grown in DMEM glutamax (31966-047, Gibco, Thermo Fischer Scientific), supplemented with 10% Fetal Bovine Serum (10270-106, Gibco, Thermo Fischer Scientific) 100 U/mL penicillin-streptomycin (15140-122, Gibco, Thermo Fischer Scientific), at 37 °C, in a humidified incubator with 5% CO_2_. Cells in a 15 cm dish were grown to 30–40% confluency and transfected with a mixture of 1:1000 dilution of Lipofectamine2000 (11668019, Invitrogen, Thermo Fischer Scientific), 1:10 dilution of OptiMEM (51985026, Invitrogen, Thermo Fischer Scientific) and 4 µg of plasmid expressing GFP-tagged candidate RBPs, media was exchanged 3–4 h after transfection and cells were harvested after 2 days. Transfection efficiency was assessed with fluorescence microscopy on live cells and was generally around 70–90%, as well as by western blotting and blotting with an anti-GFP mouse antibody (11814460001; Roche). Proteins on the membrane were also visualized by Ponceau S 0.1% (P7170-1L, Sigma-Aldrich) staining. All vectors expressing GFP-tagged candidate RBPs were ordered from GeneScript. Each of the following open reading frames was clones into a pcDNA3.1(+)-C-eGFP vector backbone: NM_016783.4 (*PGRMC1*), NM_027558.1 (PGRMC2), NM_138602.4 (*PRAF2*), NM_153457.7 (*RTN1*), NM_022030.3 (*SV2A*), NM_010239.2 (*FTH1*), NM_026479.4 (*ZCCHC10*).

### GFP-based immunoprecipitation

Immunoprecipitation was done using GFP-Trap_MA magnetic beads (gtma-20, Chromotek) according to the manufacturer’s instruction. Briefly, transfected HEK293T cell pellets were lysed (50 mM Tris-HCl, pH 7.5, 400 mM NaCl, 1 mM EDTA, 1% NP-40, 0.10% Na-deoxycholate, 25 U/mL benzonase (E1014, Sigma Aldrich) and 1 tablet per 10 mL cOmplete mini EDTA-free protease inhibitor cocktail (4693116001; Roche)), sonicated at amplitude 70% (Sonics) for 20 s and spun down at 13,000 × *g* for 10 min at 4 °C. Lysate containing 2 mg of protein (measured by Bradford assay) per sample was added to the beads and incubated for 2 h on rotator at 4 °C. Beads were washed once with lysis buffer and twice with washing buffer (50 mM Tris-HCl, pH 7.5, 150 mM NaCl, 1 mM EDTA, 1% NP-40) Samples were eluded in NuPAGE® LDS Sample Buffer (4X) (NP0007, Novex, Thermo Fischer Scientific) and analysed on a Western Blot, blotting with an anti-GFP mouse antibody (11814460001; Roche). Proteins on the membrane were also visualized by Ponceau S 0.1% (P7170-1L, Sigma-Aldrich) staining.

### Western blot analysis

Western blotting was done using standard protocols, with the following antibodies. RBM5 was visualized with mouse monoclonal anti-LUCA15 antibody (200 μg/mL; sc-515419, Santa Cruz), with a dilution of 1:1000 in 5% bovine serum albumin (BSA). GFP was visualized with the anti-GFP mouse antibody (11814460001; Roche), with a dilution of 1:1000 in 5% BSA. GAPDH was visualized with the Rabbit polyclonal to GAPDH (ab9485; Abcam) antibody, with a dilution of 1:1000 in 5% BSA.

### Cross-linking and immunoprecipitation (CLIP)

CLIP experiments were done following a protocol previously published^39^ and with the parameters described below. For CLIP on selected candidates, HEK293T cells were transfected with vectors expressing GFP-tagged proteins and cultured as described above. The cell pellet of one confluent 15 cm dish was used for each sample/condition and lysed in 1 mL PXL lysis buffer. Precipitation of cross-linked GFP-tagged RBP candidate–RNA complexes was done using GFP-Trap_MA magnetic beads (gtma-20, Chromotek): 15 µL per sample. RNase A treatment was performed with addition of 10 µL of 1:10, 1:100 or 1:1000 dilutions of stock solution (Affymetrix, P/N:70194Y). Protocol was carried out until the exposure of the radioactive SDS-PAGE. Phosphoimaging was done on a Typhoon FLA 7000 biomolecular imager.

For RBM5-CLIP, R6/2 transgenic and WT mouse brain samples were harvested, homogenized and cross-linked as described for pCLAP. For each replicate, half a brain was lysed in 6 mL PXL lysis buffer and 1 mL of the lysate per sample was incubated with beads coupled to anti-RBM5 antibodies AbR and AbM or non-specific (IgG) antibodies. Antibody-coupled beads were prepared as follows: 50 μL of Protein A Dynabeads (ThermoFischer Scientific, 10002D) per sample were washed three times with cold PBS 0.02% Tween. For AbR, beads were then re-suspended in cold PBS 0.02% Tween and 7.5 μL of rabbit polyclonal anti-RBM5 antibody (HPA018011; Merck/Sigma) and incubated on a rotator overnight at 4 °C. For AbM, beads were first incubated with 31.25 μL of a bridging antibody AffiniPure Rabbit Anti-Mouse IgG, Fcγ fragment specific (Jackson Immunoresearch, 315-005-008) in PBS 0.02% Tween, followed by rotation at room temperature for 30 min and washed three times with PBS 0.02% Tween. Then beads were re-suspended in PBS 0.02% Tween and 7.5 μL of mouse monoclonal anti-LUCA15 (200 μg/mL, sc-515419, Santa Cruz) and incubated overnight on a rotator at 4 °C. For the non-specific (IgG) control, beads were prepared as for AbM but without the addition of the mouse monoclonal anti-LUCA15 antibody. The day after, all beads were washed three times with ice-cold 1X PXL buffer prior to the addition of the mouse brain lysates. RNase A treatment was performed with addition of 10 μL of a 1:100 dilution of stock solution (Affymetrix, P/N:70194Y). Based on phosphoimager results, membrane pieces containing RNA-RBM5 complexes around 130 kDa (Supplementary Fig. 6) were cut for each sample and RNA isolated for multiplex library preparation as described previously^101^. A total of 18 libraries was generated, pooled and sequenced on an Illumina MiSeq sequencer aiming for a total depth of 30 million single end reads (1 × 150 bp). Sequencing reads were processed (Trimmomatic: standard parameters^102^, mapped to the mm10 mouse reference genome using bowtie and PCR duplicates collapsed using tools developed in-house or found in galaxy^103^. Mapped reads were then clustered, followed by quantification of the number of reads for each region for each sample. Clusters were then annotated, and their size normalized to the sample size before subsequent analyses. Clusters with a two-fold enrichment in both AbR and AbM samples compared to IgG samples were included in the list of RBM5-binding sites. Differences in RBM5 binding to transcripts were also defined by an at least two-fold change in reads over the binding sites between HD and WT samples with both antibodies. Significance of overlaps was assessed by Fisher’s exact test.

### RNA-seq analysis

Mouse brain tissue from the same animals as for the RBM5-CLIP analysis was used for RNA-seq analysis. Total RNA was extracted from a small fraction of each of the non-cross linked brain halves using the RNeasy micro kit (Qiagen). Sequencing libraries were constructed from 200 ng of total RNA using NEBNext rRNA Depletion Kit (Human/Mouse/Rat), stranded NEBNext Ultra II Directional RNA Library Prep kit for Illumina and NEBNext Multiplex Oligos for Illumina (NEB). Libraries were sequenced on a NextSeq500 Illumina aiming for a depth of 10 million paired end reads (2 × 75 bp) per sample. Sequencing reads were trimmed to remove poor quality sequence and adaptors using Trimmomatic^102^ using parameters LEADING:3 TRAILING:3 SLIDINGWINDOW:4:15 MINLEN:33. Sequence reads were mapped to the mm10 mouse reference genome using RNA STAR (v2.7.3) following the two-pass alignment protocol^104^. BAM files were imported in SeqMonk (v 1.45.4, Babraham Institute) where data was assessed for data integrity (RNA-Seq QC pipeline), and quantified using the RNA-Seq quantitation pipeline to generate raw counts to mRNA and setting library type to ‘opposing strand specific’. Differentially expressed genes were calculated using SeqMonk edgeR^105^ via the ‘statistical test on replicated data’ script as generated via SeqMonk. For identification of differentially expressed exons and introns we quantified reads over each exon and intron in SeqMonk and then performed edgeR analysis. Transcripts that had changes in 100% of their exons or introns were excluded from further analysis as this indicated a differential expression of the whole transcript.

### RNA isolation, cDNA library preparation and RT-PCR

For PCR validation of alternative splicing, WT and R6/2 HD brains were collected from animals, independent from the ones used for RBM5-CLIP and RNA-Seq. In this case, brain halves were not dissociated as described before but immediately snap-frozen. Total RNA was then extracted with Trizol and RNA clean and concentrator-25 kit (Zymo), where a separate brain was used for each replicate. Single-strand cDNA was synthesized using 1 μg total RNA and the Superscript III First-Strand Synthesis Supermix. PCR was performed from 1:10 cDNA dilution with Q5 Hot Start High-Fidelity 2X Master Mix (NEB) and the oligonucleotides (0.2 μM) described below. PCR conditions were similar for all sets of oligonucleotides: 2 min 98 °C, 30× (30 s 98 °C, 30 s 60 °C, 1 min 72 °C), 5 min 72 °C. Samples from different animals were processed in parallel from RNA extraction to PCR. Similar volumes of PCR reactions for each sample were loaded and run in parallel on TAE 2% agarose gels with gel red (Biotum) and 100 bp marker (NEB) before imaging with UV imager (Biorad).

The following oligonucleotide forward (fw) and reverse (rev) primer pairs were used for RT-PCR of selected candidates (see also Fig. 5): *Adamts10* (fw: TCATCCTGCTCACAGAGGA; rev: CCACGCCATCGTGATTCAT); *Atrx* (fw: GAGACCAAAGAACCGTTAGT; rev: GGGCAAGAATACATCCTGAA; *Ptprn* (fw: TTCTACCCTCTTGACCCTG; rev: ATCGGCTCCTCCAACATTTA).

Abundance of the PCR products were quantified using the gel analysis pipeline in ImageJ (https://imagej.nih.gov/) on colour-reversed gel pictures taken after short exposure, whereas the images shown in figures were taken after longer exposure. Significance of differences between the PCR products from WT and HD samples was assessed using a standard two-tailed *t* test in Prism assuming equal variance. The average mean and standard deviation (error bars) for band intensities for each genotype can be seen on the graph and in the Source Data file. Significance threshold for *p* values was set to 0.05. *p* Values for the comparisons (HD vs WT) are as follows: Atrx band 1 *p* = 0.0101, band 2 *p* = 0.371; Ptprn band 1 *p* = 0.024, band 2 *p* = 0.856; Clasrp band 1 *p* = 0.001, band 2 *p* = 0.063; Adamts10 band 1 *p* = 0.016, band 2 *p* = 0.239, band 3 *p* = 0.056, band *p* = 0.632.

### Overexpression and knock-down of *RBM5* in human LGE-patterned neurons

hESC (RC17 from Roslin Cells, hPSCreg RCe021-A) were maintained in Laminin-521 (1 μg/cm^2^, Biolamina, #LN521-02) diluted in PBS+/+ (ThermoFisher, #14040091) and passaged at described previously^106^. Differentiation towards LGE-patterned neurons was carried out using a modified version of a previously published protocol^106^. More precisely, 1 day of differentiation 0, 70–90% confluent hESC were dissociated using 0.5 mM EDTA (Thermo Fisher Scientific, #15575020) for 7 min at 37 °C. Then, cells were collected and resuspended to a single-cell suspension in wash medium containing 9.5 mL DMEM/F12 (ThermoFisher, #10565018) and 0.5 mL KnockOut Serum Replacement (ThermoFisher, #10828028) and counted using a Neubauer counting chamber. For neural induction, hESC were plated on Laminin-111 coated plates (2 μg/cm^2^, Biolamina, #LN111-02) at a density of 25.000 cells/cm^2^ in N2 medium supplemented with 10 μM SB413542 (Miltenyi, #130-106- 543) and 100 ng/mL Noggin (Miltenyi, #130-103-456) from day 0 to day 9. 10 μM Y-27632 (ROCK inhibitor, Miltenyi, #130-106-538) was added to the media for the first 48 h to increase survival. N2 medium was changed every two days until day 9 of differentiation. At day 9, media was replaced with N2 medium plus Activin A (25 ng/μL, R&D Systems, #338-AC-010).

At day 11, early neural progenitors were dissociated in 75 μL/cm^2^ accutase (ThermoFisher, #A11105-01) for 7 min at 37 °C collected and resuspended to a single-cell suspension in wash medium and counted as previously described. Cells were replated at a cell density of 800.000 cells/cm^2^ in Laminin-111 (2 μg/cm^2^, Biolamina, #LN111-02) coated cell culture plates in B27 media supplemented with 0.2 mM ascorbic acid (Sigma-Merck, #A4403-100MG), 20 μg/mL BDNF (Miltenyi, #130-096-286) and Activin A (25 ng/μL, R&D Systems, #338-AC-010), adding Y-27632 for 24 h. Media was changed every second day until day 16.

At day 16, progenitors were dissociated in accutase and counted as described before. Progenitors were transduced with custom made lentiviral (LV) particles containing either knock-down and overexpression vectors for RBM5 and their respective controls (VectorBuilder Inc, Catalogue #: LVM(VB010000-0009mxc)-C, LVM(VB010000-9298rtf)-C, P220412-1015udg and P220412-1015udg). To do so, progenitors were incubated in an Eppendorf tube with LV particles at a multiplicity of infection (MOI) of 2 for 30 min at 37 °C in 200 μL of maturation media supplemented with 10 μM Y-27632. Then, cells were plated at a density of 700,000 cells/cm^2^ into Laminin-521 coated (2 μg/cm2, Biolamina, #LN521-02) cell culture plates. 4–6 h later, medium amount was top up to 400 μL/cm^2^. Maturation media consisted of B27 medium supplemented with 0.2 mM ascorbic acid (Sigma-Merck, #A4403-100MG), 20 μg/mL BDNF (Miltenyi, #130-096-286), 500 μM dibu-tyryl-cAMP (Sigma-Merck, #D0627-1G) and 1 μM DAPT (Miltenyi, #130-110-489). 10 μM Y-27632 (ROCK inhibitor, Miltenyi, #130-106-538) was added to the medium for 24 h after replating. 24 h later, medium was replaced to remove lentiviral particles from the cells and then medium (B27 + ascorbic acid, BDNF, db-cAMP and DAPT) was changed every 2 days until day 26 of differentiation, when cells were collected and lysed in 350 μL of RLT buffer (QIAGEN, #74034) containing 0.5 mM beta-mercaptoethanol (ThermoFisher, #31350010) and subsequently stored at −80 °C.

### RNA isolation, cDNA preparation and RT-qPCR on neurons

For RNA isolation, all samples were processed on a QIAcube using the RNeasy plus micro kit (Qiagen, #74034) following the instructions from the manufacturer. cDNA synthesis from up to 1 μg of RNA was carried out using the Maxima first strand cDNA synthesis kit (ThermoFisher, #K1642). The cDNA was subsequently diluted in EB buffer (Qiagen, #19086) and stored at −20 °C until RT-qPCR analysis.

The gene expression profiles of the LGE-patterned neurons were analysed by RT-qPCR. To do so, 0.4 μL cDNA, 1,6 μL of 0.96 μM primer mix (see sequences below) and 2 μL SYBR green (Roche, #04887352001) were mixed using the iDOT liquid handler and analysed by RT-qPCR on a Light Cycler 480 II instrument (Roche, #05015243001) using a 40x cycle two-step protocol with a 60 °C, 60 s annealing/elongation step and a 95 °C 30 s denaturation step. The average threshold cycle (CT) values of technical duplicates were used to calculate the relative gene expression using the comparative CT method (∆∆CT)^107^. For each target gene, the fold change of the differentiated neurons was calculated relatively to undifferentiated hESC reference and the expression of the two housekeeping genes *ACTB* and *GAPDH*. The following primer pairs were used for the RT-qPCR: *ADAMTS10* intron (fw: TGCGGCACACTAGGCCTG; rev: TCTCGTGGGCAATGGTGAA); *ADAMTS10* exon (fw: TGCAAGGATGTGAACAAGGT; rev: GCAGGTTTTGCAGCACATCT); *ADAMTS10* normalization (fw: ATCCCCACATGTCCGTCTT; rev: TCTCGTGGGCAATGGTGAA); *ATRX* intron (fw: GATGTGTTTATGTAAAGGAAGT; rev: AGCAATCCCACATAAACTGAA); *ATRX* exon (fw: TCAAGTAGATGGTGTTCAGTT; rev: GGGCAAGAATGCATCCTGA); *ATRX* normalization (fw: GTGTTCAGCGAATACTTATGA; rev: GCTTTGGCATCATGAGGTG); *PTPRN* intron (fw: CCCTCTGCCTGGACACTT; rev: GAGGCTGGCCGCTGTGCT); *PTPRN* exon (fw: CCTGGTGTCGGAGCACAT; fw: CAGCTGAGGAAGTGGAACT); *PTRPN* normalization (fw: AACCCCTCCCGGATTTCTT; rev: GAGGCTGGCCGCTGTGCT); *ACTB* (fw: ATGTGGCCGAGGACTTTGATTG; rev: ATGGCAAGGGACTTCCTGTAAC); *GAPDH (fw:* TTGAGGTCAATGAAGGGGTC; rev: GAAGGTGAAGGTCGGAGTCA); *GAD1 (fw:* CTCCTGGGGGCGCCATATCCAA; rev: CCAGTTTAGGCACAGCCGCCAT); *MAP2* (fw: CCGTGTGGACCATGGGGCTG; rev: GTCGTCGGGGTGATGCCACG); *BCL11B* (CTIP2) (fw: CTGGAGATAGAGGAGCCAAGTG; rev: TAAAAACCAGGATGTCCCCCAA).

Significance of differences between the PCR products from WT and HD samples was assessed using a standard two-tailed t-test assuming equal variance. The average mean and standard deviation (error bars) for band intensities for each genotype can be seen on the graph and are provided in the Source Data file. Significance threshold for *p* values was set to 0.05. *p* Values for the comparison of control OE and *RBM5* OE are the following: *RBM5*—0.0203; *ATRX*—0.0426; ADAMTS10—0.0049; *PTRPN*—0.2474. *p* Values for the comparison of control OE and *RBM5* OE are the following: *RBM5*—0.0011; *ATRX*—0.0313; *ADAMTS10*—0.0549; *PTRPN*—0.0363.

### RBM5 AP-MS from WT and HD R6/2 transgenic mouse brain tissue

In all, 25 µL of protein-G Dynabeads (10003D; Thermo Fischer) were washed three times with PBS and after re-suspension in PBS, 20 µL of polyclonal rabbit antibody (HPA018011; Merck/Sigma), 10 µL of mouse monoclonal anti-LUCA15 antibody (200 μg/mL; sc-515419, Santa Cruz) or no antibody (negative control) was added and incubated overnight on a rotator at 4 °C. Beads were washed three times with PBS and the antibodies were cross-linked to the protein-G beads by incubation in 19 mM Dimethyl pimelimidate dihydrochloride (D8388, Sigma Aldrich) in 20 mM sodium borate (pH 9.0) for 30 min at room temperature on a rotator. Beads were washed three times with ice-cold PBS and non-cross-linked antibodies eluded with 0.5 M glycine (pH 2.5) on a rotator for 10 min. Beads were washed three times with PBS and three times in lysis buffer prior to addition to brain tissue lysates. Homogenized WT and HD mouse half-brains (homogenized as for pCLAP) were re-suspended in lysis buffer (50 mM TrisHCl (pH 7.5), 150 mM NaCl, 0.5% NP-40, 0.1% Na-deoxycolate, 5% Glycerol, 1 mM, 5 mM β-glycerophosphate, 1 mM sodium fluoride, 1 mM sodium orthovanadate, 125 U/mL of Benzonase (E1014, Sigma Aldrich) and 1 tablet per 10 mL cOmplete mini EDTA-free protease inhibitor cocktail (4693116001; Roche)) and lysed for 1 h on a rotator at 4 °C and spun down at 4700 × *g* for 15 min at 4 °C. Protein concentration was measured by Bradford assay using Quick Start Bradford 1× Dye Reagent (#5000205; Bio-Rad) and lysate containing 2 mg of protein was used for each Co-IP replicate. Samples were pre-cleared by incubation with protein G-dynabeads for 1 h at 4 °C on a rotator and the supernatant was incubated with the beads cross-linked to AbM, AbR or no antibody for 4 h on a rotator at 4 °C. The Co-IP samples were washed with lysis buffer without benzonase or protease inhibitors three times for 10 min on a rotator at 4 °C. Samples were washed with PBS three times to remove detergent and salts and re-suspended in digestion buffer (50 mM ABC (pH 8.0), 10 mM CAA, 5 mM TCEp). 2.5 µL of trypsin (0.5 µg/µL; Sigma) and LysC (0.5 µg/µL; Wako) was added to each sample and incubated for 1 h at 4 °C on a shaker at 1200 rpm, followed by overnight incubation at 37 °C on a shaker at 350 rpm. The supernatant with the digested peptides was moved to a new tube, acidified, desalted and concentrated on C18 StageTips as described for the pCLAP experiments.

Samples were eluded from StageTips in 2 steps with 40% ACN and 0.5% acetic acid, concentrated and acetonitrile was evaporated using a Vacufuge at 45 °C until the acetonitrile had evaporated and 5% ACN and 0.1% TFA was added to each sample to bring sample volume to 30 µL. 1/6 of each sample was separated on an EASY-nLC 1200 System (Thermo Fischer) with 15 cm long in-house pulled and packed column, filled with ReproSil-Pur 120 C18-AQ, 1.9 µm (r119.aq; Dr. Maisch). Peptides were separated out on a 80-min low pH gradient with a mobile phase of 0.1% Formic acid (1.00264.1000, VWR) and an Acetonitrile concentration gradient ranging from 0 to 80%. From the tip of the column, samples were ionized at 270 °C and 2 kV and analysed on an Orbitrap Exploris 480 Mass Spectrometer (Thermo Fischer). The full scan was done in positive mode with Orbitrap resolution of 120,000, scan range 300–1750, AGC target Custom, normalized AGC target 200%. For each full scan 9 fragment scans were selected, with an intensity threshold of 2.5E + 5, included charge states 2–6, dynamic exclusion of 60 s and a mass tolerance of 20 ppm. Fragment scans were done with an isolation window of 1.3 *m*/*z*, Orbitrap resolution of 45,000, HCD collision energy of 25%, AGC target custom, normalized AGC target 200% and maximum injection time mode auto. Analysis of the MS data was done as described for the pCLAP samples, with the difference that ‘match between runs’ was turned off.

### Gene Ontology (GO) term analysis of CLIP, RNA-seq and Co-IP samples

GO term analysis in this study was done using GeneCodis (https://genecodis.genyo.es/)^98^ and PANTHER^99^. The only exception is the RBM5 proteins interactome study, where AmiGO (http://geneontology.org/) and PANTHER^99^ was used, since GeneCodis was unavailable at that point in time. All shown *p* values are using the multiple hypothesis corrected values provided by these tools.

### Reporting summary

Further information on research design is available in the Nature Portfolio Reporting Summary linked to this article.

## Supplementary information

Supplementary Information

Description of Additional Supplementary Files

Supplementary Data 1

Supplementary Data 2

Supplementary Data 3

Supplementary Data 4

Supplementary data 5

Supplementary Data 6

Reporting Summary

Peer Review File

## Data Availability

The mass spectrometry proteomics data generated in this study have been deposited to the ProteomeXchange Consortium via the PRIDE partner repository with the dataset identifier PXD029227. CLIP-seq and RNA-seq data can be accessed via the following GEO accession number: GSE187445. Source data are provided with this paper.

## Source data

Source Data
