# Supplementary material for: Characterising the RNA-binding protein atlas of the mammalian brain uncovers RBM5 misregulation in mouse models of Huntington’s disease
Source: Nat Commun. 2023 Jul 19;14:4348. doi: 10.1038/s41467-023-39936-x (PMC10356804; doi:10.1038/s41467-023-39936-x)
Supplement: Supplementary file 1 — Supplementary Information [file 41467_2023_39936_MOESM1_ESM.pdf]

# Supplementary material for

# Characterising the RNA-binding protein atlas of the mammalian brain uncovers RBM5 misregulation in mouse models of Huntington's disease

Meeli Mullari[1,9]*, Nicolas Fossat[2,3,9], Niels H. Skotte[1,8], Andrea Asenjo-Martinez[4], David T. Humphreys[5], Jens Bukh[2,3], Agnete Kirkeby[4,6], Troels K. H. Scheel[2,3,7], and Michael L. Nielsen[1,] *

[1]Proteomics program, Novo Nordisk Foundation Center for Protein Research, Faculty of Health and Medical Sciences, University of Copenhagen, Denmark

[2] Copenhagen Hepatitis C Program (CO-HEP), Department of Immunology and Microbiology, Faculty of Health and Medical Sciences, University of Copenhagen, Denmark

[3] CO-HEP, Department of Infectious Diseases, Copenhagen University Hospital, Hvidovre, Denmark

[4]The Novo Nordisk Foundation Center for Stem Cell Medicine (reNEW) and Department of Neuroscience, University of Copenhagen, Denmark

[5]Victor Chang Cardiac Research Institute, Darlinghurst, NSW 2010, Australia

[6] Wallenberg Center for Molecular Medicine (WCMM) and Department of Experimental Medical Science, Lund University, Sweden

[7]Laboratory of Virology and Infectious Disease, The Rockefeller University, New York, NY, USA

[8]Department of Drug Design and Pharmacology, Faculty of Health and Medical Sciences, University of Copenhagen, Copenhagen, Denmark

[9]These authors contributes equally: Meeli Mullari and Nicolas Fossat

*Correspondence should be addressed to: mullari.meeli@gmail.com (M.M.)
michael.lund.nielsen@cpr.ku.dk (M.L.N)

Contents:

Supplementary Figure legends

Supplementary figures

# Supplementary figure 1

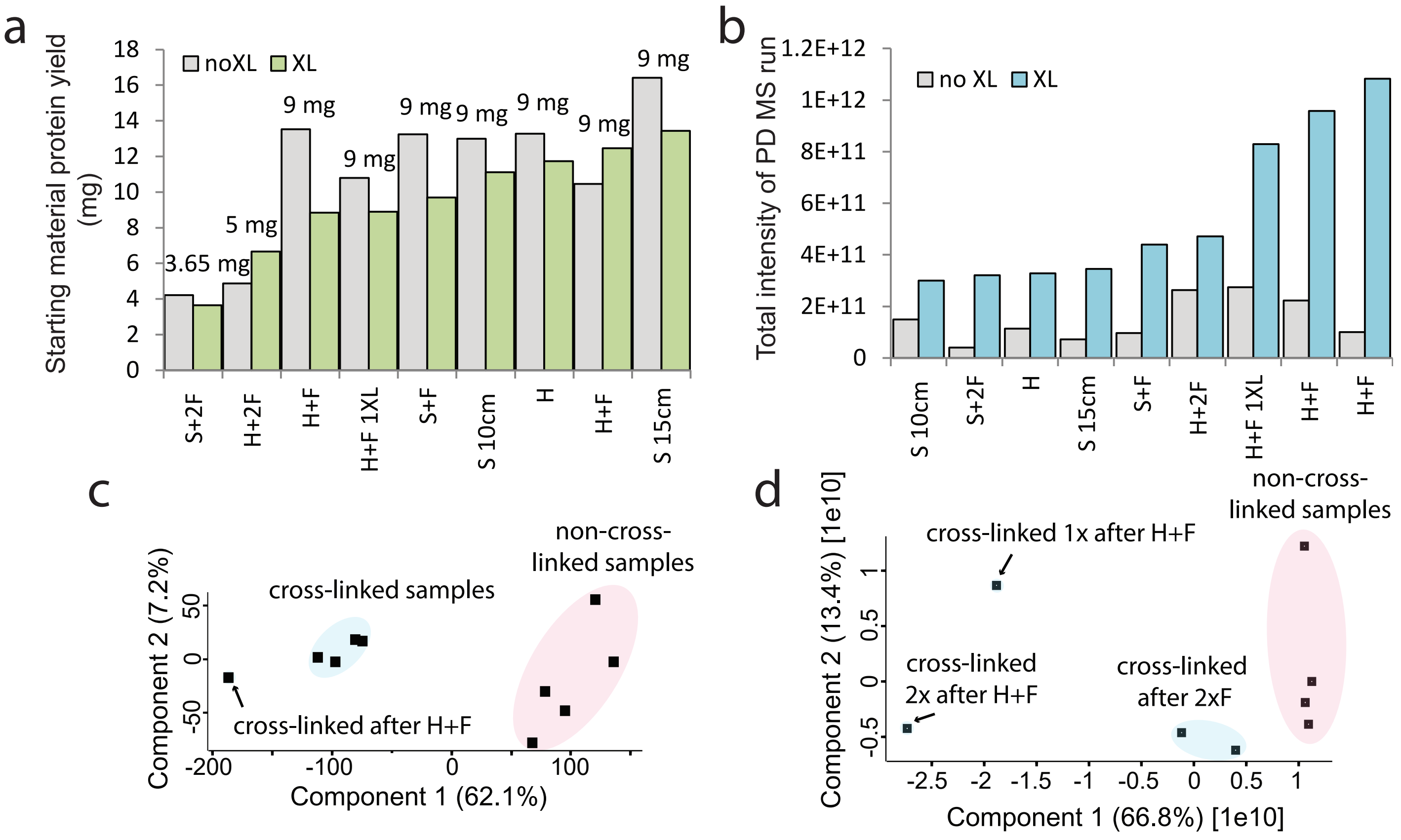


**Supplementary figure 1. Optimization of tissue dissociation for the efficient cross-linking of RNA-protein complexes in mouse brain tissue. (A)** Protein yield in mg from half a brain after tissue homogenization, cross-linking and lysis. Above each pair of XL and noXL sample the protein amount used for input for pCLAP is shown. **(B)** Total intensity of XL and noXL pCLAP MS runs for each homogenization method **(C-D)** PCA plots showing cross-linked and non-crosslinked samples of pCLAP-MS samples for all homogenization methods. Cross-linked sample – blue shading, non-cross-linked samples – pink shading. Homogenization methods tested on mouse brain tissues – S 10cm: pressed through tea sieve with a syringe plumber and cross-linked 2x with UV on the surface of a 10cm cell-culture dish in PBS (all others cross-linked on the area of 15 cm plates and also in PBS). S+2F: pressed through tea sieve and 2x through a 50uM filter with a syringe plumber, cross-linked 2x. H: tenbroeck homogenizer and cross-linked 2x. S 15cm: pressed through tea sieve with a syringe plumber and cross-linked 2x. S+F: pressed through tea sieve and 1x through a 50uM filter with a syringe plumber and cross-linked 2x. H+2F: tenbroeck homogenizer and pressed 2x through a 50uM filter with a syringe plumber and cross-linked 2x. H+F 1XL: tenbroeck homogenizer and pressed through a 50uM filter with a syringe plumber and cross-linked 1x. H+F: tenbroeck homogenizer and pressed through a 50uM filter with a syringe plumber and cross-linked 2x (tested and shown twice).

# Supplementary figure 2

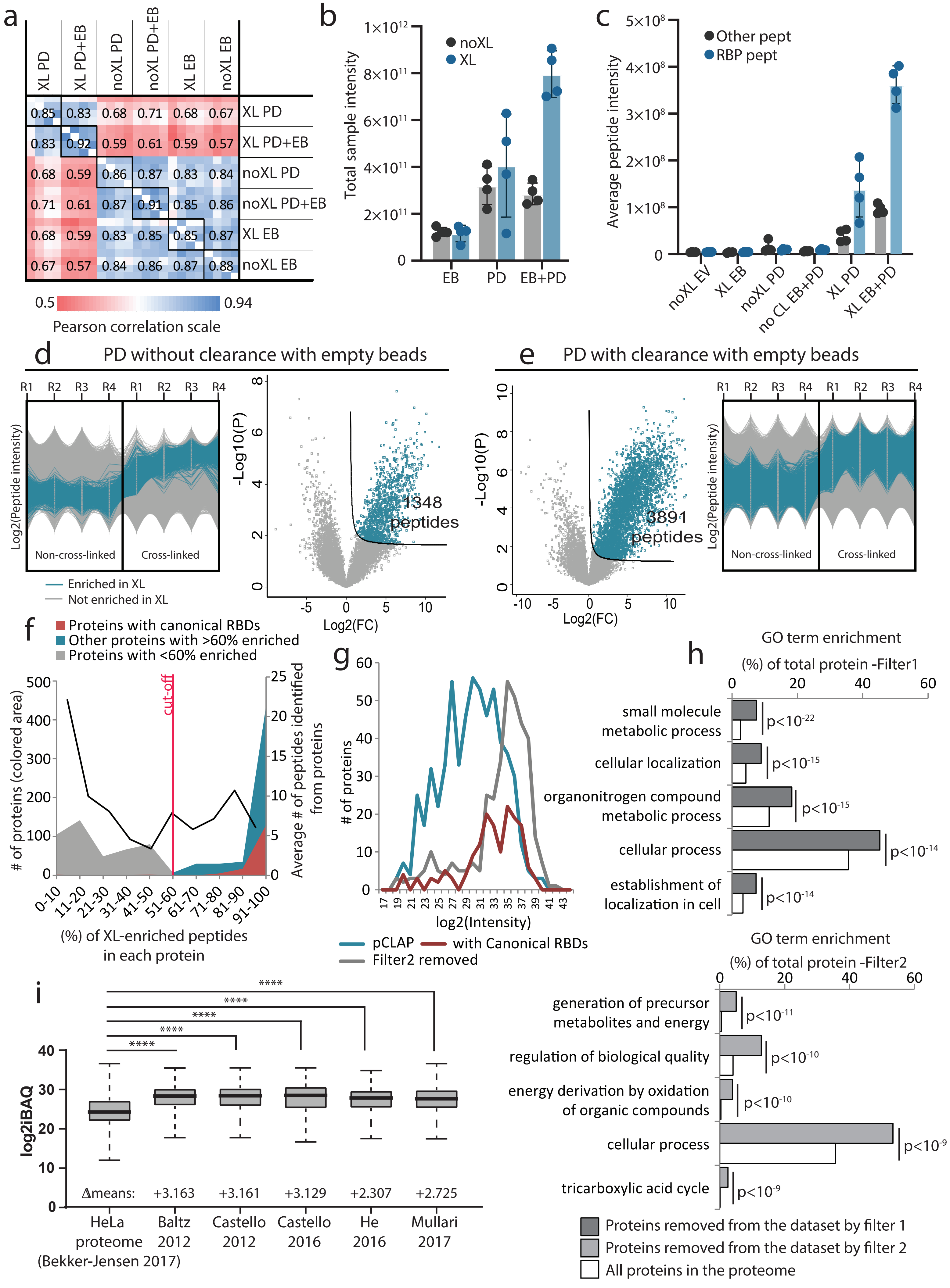

**Supplementary figure 2. (A)** Heat map representation of pCLAP data Pearson correlations between all samples. **(B)** Total peptide intensities averaged between four replicates for cross-linked (XL) and non-cross-linked (noXL) samples. **(C)** Average intensities for peptides from proteins with known canonical RBDs and other peptides in all samples. For all dot plots n=4 biologically independent animals, data are presented as mean values +/-SD and source data are provided as a Source Data file. **(D-E)** Volcano plots showing enrichment between XL and noXL samples for pCLAP experiments with and without pre-clearance. Peptides marked in blue are significantly enriched in XL samples. On the corresponding profile plots, each line represents one peptide identified in the pCLAP sample and shows the intensity of the peptide in the four non-cross-linked samples compared to the four cross-linked samples. Blue lines represent peptides significantly enriched in XL samples from the volcano plot. Many of the values in the non-cross-linked samples are imputated and were not really detected in these samples. Significance was calculated using a two-sided t-test with multiple-testing correction. **(F) Filtering step 2:** Analysis showing distribution of proteins in the sample based on percentage of peptides enriched in the cross-linked samples. Red line separates proteins with >60% of peptides identified being enriched in cross-linked samples, which were included in the list of RBPs identified in this study (blue and red area). Distribution of proteins with canonical RBDs (PurA, CSD, RRM, KH, PUF, RNase_T, dsrm, La, DEAD, Helicase_C, S1) is shown in red. The black line depicts the average number of peptides identified for the proteins in each specific percentage bin. **(G)** Abundance distribution of proteins included in the RBP list after filtering step 2 (blue line), those removed as contaminant peptides in filtering step 2 (grey line) and proteins with canonical RBDs identified in the pCLAP experiment (red line). Protein abundances from Sharma et. al.[1]. **(H)** GO term enrichment analysis for proteins removed from the final pCLAP identified RBP list by filtering steps 1 and 2. Significance was calculated using a one-tailed Fischer's exact test with multiple-testing correction. **(I)** Abundance distributions for proteins from a deep proteome study and cell based RNA-interactome studies. "Means:" under boxplots show the upward shift of the mean for proteins identified as RBPs in cell based studies compared to total HeLa proteome[2]. The middle of the box is the mean, the box boundaries represent the 25$^{th}$ and 75$^{th}$ percentile and the whiskers reach to the minimum and maximum value of the dataset. P-value calculated using two-tailed t-test, ****p<0.0001.

# Supplementary figure 3

$$\%\ \text{of XL-enriched peptides in a protein} = \frac{\text{N peptides enriched in XL}}{\text{N total peptides identified}} \times 100\%$$

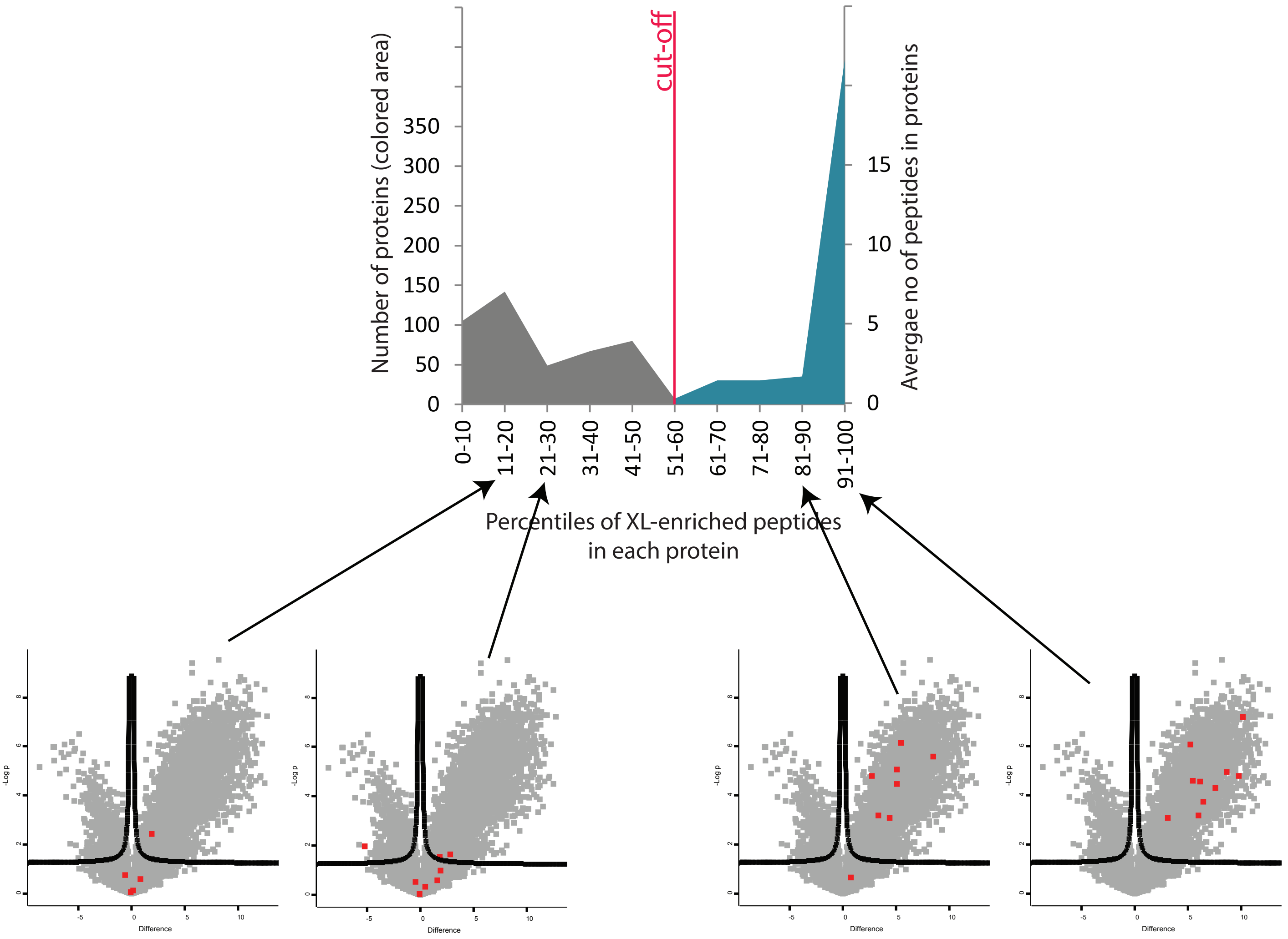


Hist1h3a 20%
Total pept: 5
XL enriched: 1
1/5*100%=20%

Hist3h2ba 28.6%
Total pept: 7
XL enriched: 2
2/7*100%=28.6%

Celf5 87.5%
Total pept: 8
XL enriched: 7
7/8*100%=87.5%

FUS 100%
Total pept: 10
XL enriched: 10
10/10*100%=100%

**Supplementary figure 3. Explanation of filter 2 used for the removal of abundance driven contaminants from the tissue-pCLAP data. Top:** Formula showing how percentage of peptides that were enriched in the cross-linked samples compared to total number of peptides in the samples per protein was calculated (XL enriched peptides in protein). N – number of peptides. **Middle:** Distribution of proteins in the dataset after applying ‘filter 1’ based on their percentage of peptides enriched in the cross-linked sample. Blue area signifies proteins that were kept in the dataset after ‘filter 2’. **Bottom:** Examples of proteins with a high and low percentage of peptides enriched in the cross-linked sample and how the percentage was calculated. Red dots mark peptides identified from the specific proteins and grey dots show all the peptides identified in the samples. Black lines mark significance based cut-off for enrichment in the cross-linked and non-cross-linked samples. Arrows show in which percentage bin the proteins fall into.

# Supplementary figure 4

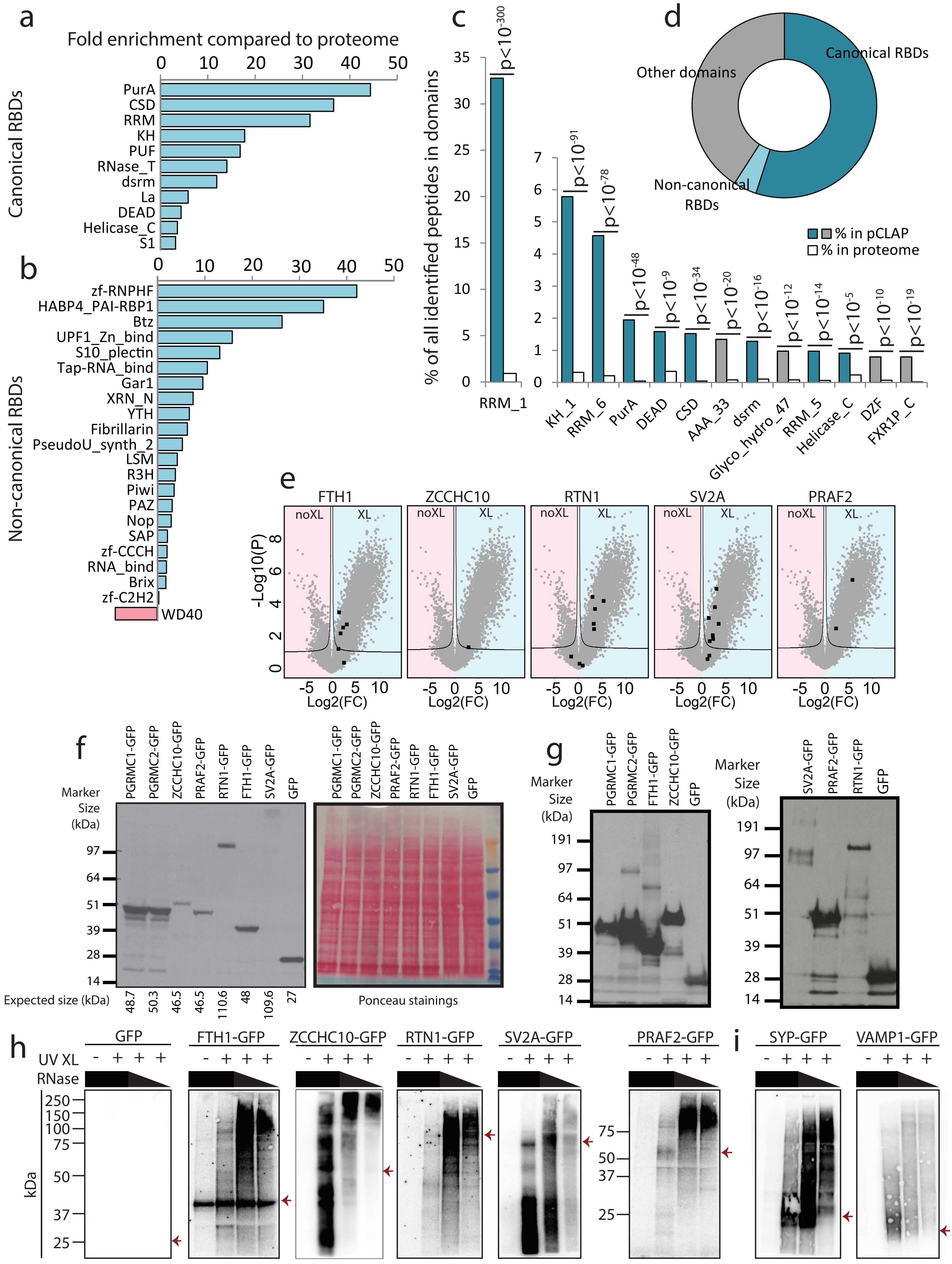

**Supplementary figure 4. (A-B)** All peptides identified by pCLAP as RNA-binding were mapped to the pfam database and enrichment of peptides originating from canonical (A) and non-canonical (B) RBDs, compared to a previously published mouse brain proteome[1] is shown in blue bars. **(C)** Percentage of pCLAP data peptides mapping to RBDs shown is compared to mapping of peptides from the brain proteome (one-tailed Fischer's exact test with FDR correction). **(D)** Total percentage of peptides annotated to map to canonical RBDs (darker blue), non-canonical RBDs (lighter blue) and other domains (grey). **(E)** Volcano plot of the pCLAP experiment comparing WT and HD samples, where black dots signify peptides identified for candidate RBPs named above the plot, chosen for validation of RNA-binding ability. Significance was calculated using a two-tailed t-test with multiple-testing correction. **(F)** Western blot showing the expression of exogenously expressed GFP-tagged candidate RBPs in transfected HEK293T cell lysates; staining with antibody against GFP on the left, Ponceau stain on the right. Expected sizes of the expressed GFP-tagged construct is shown under the WB. **(G)** Western blot of GFP-based pull down samples from the same transfected HEK293T cells; staining with antibody against GFP. **(H)** CLIP analysis from transfected HEK293T cells, where GFP-based pull downs were used to enrich for candidate RBP-GFP-RNA complexes. First lane shows the non-cross-linked control and the rest of the samples show the change in the size of the cross-linked protein-RNA complexes upon addition of increasing amounts of RNase. Each lane of each CLIP experiment represents a replicate of independently grown cells. **(I)** CLIP experiment for GFP-tagged SYP and VAMP1, presented and performed as in (H).

# Supplementary figure 5

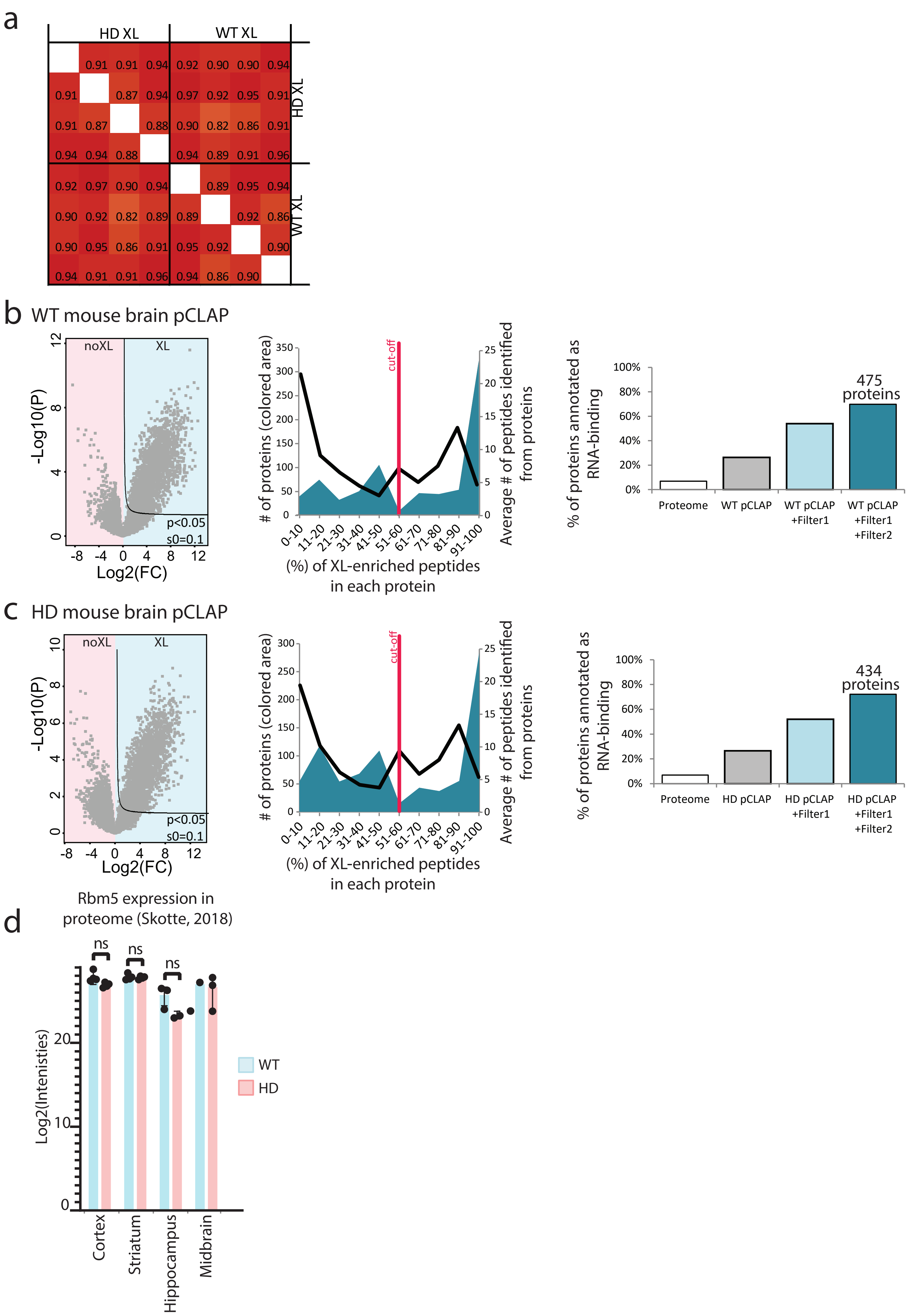

**Supplementary figure 5. (A)** Pearson correlation matrix for pCLAP replicates from WT and HD samples. **(B-C)** Filtering steps 1 and 2 done on the WT and HD samples and the number of RBPs among identified proteins before and after filtering. Also see legends for Figure 1C-D and Supplementary figure 2F. Significance for enrichment in cross-linked and non-cross-linked samples was calculated using a two-sided t-test with multiple-testing correction. **(D)** Expression of Rbm5 protein in the R6/2 mouse brain proteome[3] (two-tailed t-test was used).

# Supplementary figure 6

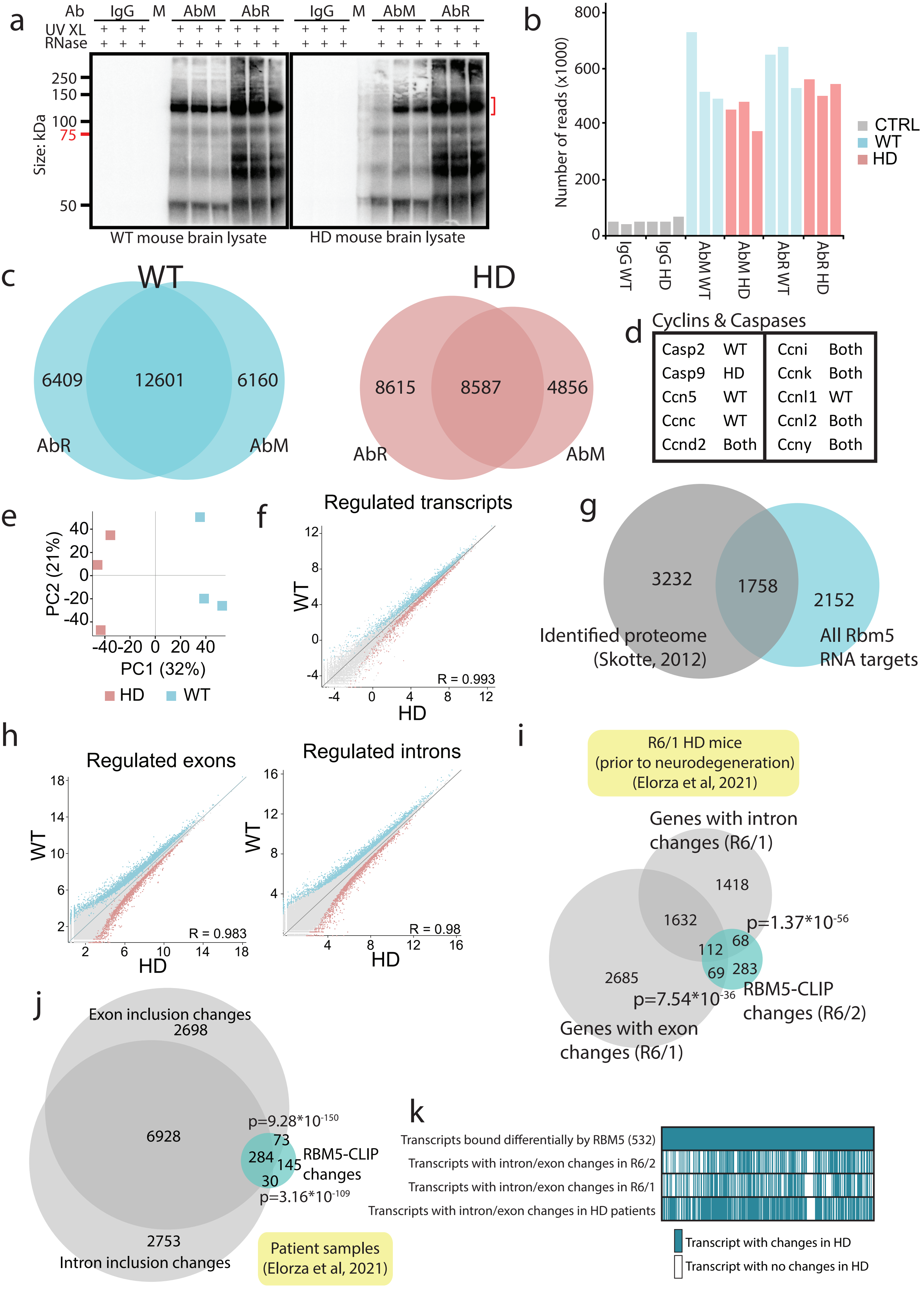

**Supplementary figure 6.** **(A)** Autoradiography detecting isotope-labelled RBM5-RNA complexes isolated with anti-RBM5 antibodies (AbM or AbR) or IgG control. Same cross-linking conditions and RNase concentrations were used for all samples. Area of the membrane containing Rbm5-RNA complexes (as indicated by red bracket) was cut-out for each sample and processed according to the rest of the HITS-CLIP protocol. **(B)** Number of reads in each CLIP sample after quality filtering, duplicate reads removal, collapsing and adapter removal. **(C)** Overlap between RBM5-binging sites (clusters) identified with each antibody from WT and HD samples. **(D)** Caspases and cyclins identified as Rbm5 RNA targets in mouse brain samples from WT, HD or Both. **(E)** PCA plot of RNA-seq data from HD and WT mouse brain tissue. **(F)** Scatter plot comparing RNA-seq data from WT and HD mouse brain tissue, where each dot represents a transcript. Blue dots represent transcripts significantly down regulated in HD and red dots significantly up regulated in HD. **(G)** Overlap between genes encoding transcripts bound by RBM5 and genes encoding all proteins identified from WT and HD in a proteomics study[3]. **(H)** Scatter plots comparing WT and HD RNA-seq samples, where each dot represents either one exon or intron. Blue dots represent introns/exons significantly down regulated in HD and red dots significantly up regulated in HD. **(I)** Overlap of RBM5-binding changes in brain tissue from the HD R6/2 mice from our study compared to transcripts where at least one exon or intron was differentially regulated between HD and WT in the RNA-seq data from brain tissue from the early symptomless R6/1 HD mice[4] (p-values calculated using one-sided Fischer's exact test and corrected for multiple testing). Transcripts where all exons or introns were regulated were excluded, since this suggest expression changes, rather than splicing changes. All analysis was done as for the R6/2 RNA-seq data. **(J)** Overlap of RBM5 binding changes in R6/2 mice and transcripts undergoing differential splicing in human HD patient brain samples from[4] (p-values calculated using one-sided Fischer's exact test and corrected for multiple testing). **(K)** Splicing changes in transcripts that are bound differentially by Rbm5 in R6/2 in R62, R6/1 and patient brain samples.

# Supplementary Figure 7

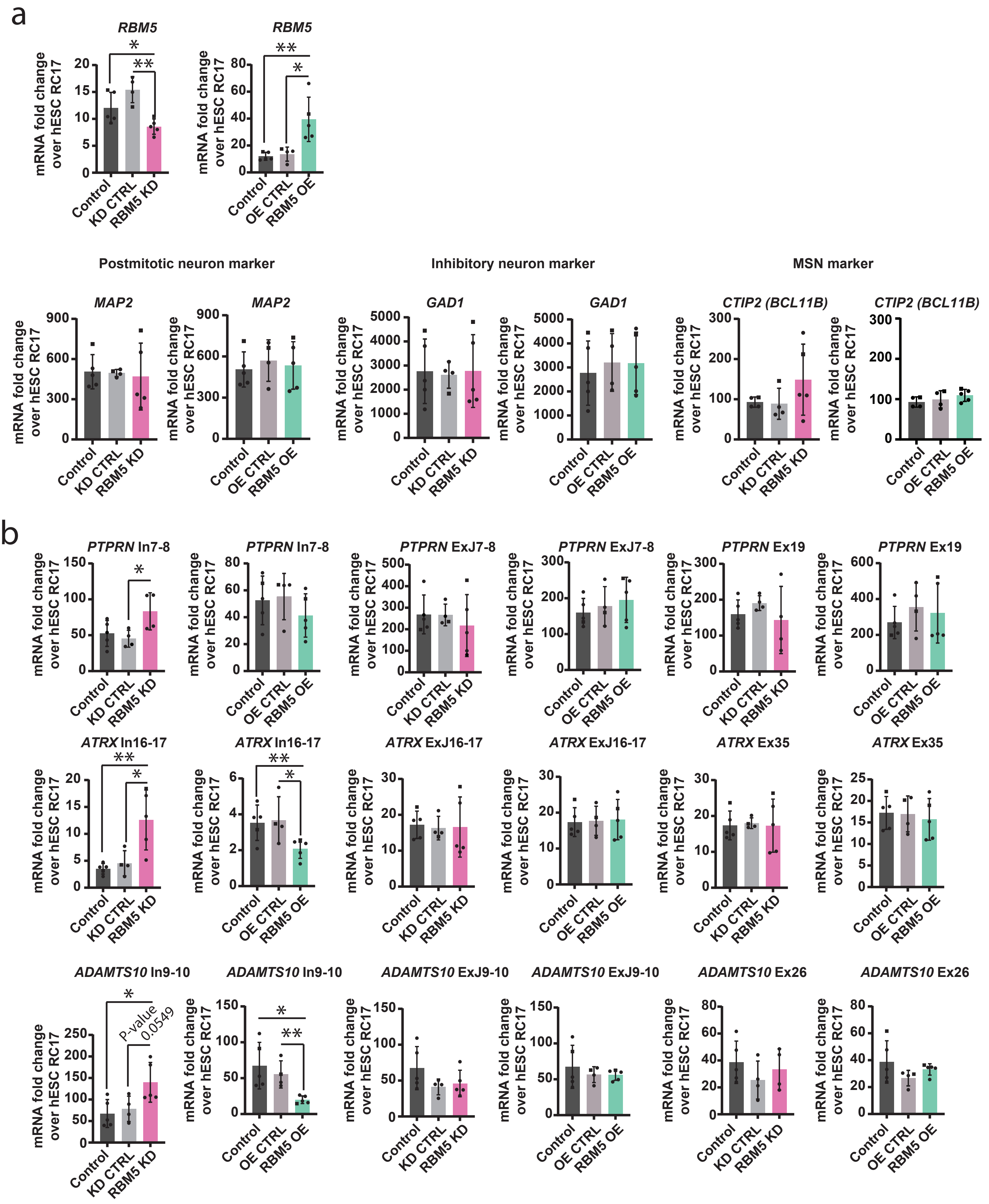

**Supplementary figure 7. qPCR analysis of RBM5 OE and KD differentiated human neurons. (A)** RT-qPCR for 4-5 independent biological replicates for human neurons differentiated from hESCs amplifying an exonic region of RBM5 and confirming *RBM5* knock down and over-expression (top) and RT-qPCR on neuronal markers confirming correct neuronal differentiation (below). For each transcript data is presented from non-transduced neurons (control), neurons transduced with control OE/KD vector and RBM5 KD/OE vector. **(B)** RT-qPCR from 4-5 independent biological replicates amplifying intronic regions (left) and two separate exonic regions (middle and right) for *ATRX, ADAMTS10* and *PTRPN* transcripts from non-transduced neurons (control), neurons transduced with control OE/KD vector and RBM5 KD/OE vector. Asterisks show significant changes and 'ns' signifies no change, *$p<0.05$ and **$p<0.01$ (n=5 biologically independent replicates for CTRL, KD and OE experiments and n=4 biologically independent replicates for CTRL KD and CTRL OE experiments, with differentiation and transduction performed separately for each replicate). Data are presented as mean values +/- SD and source data are provided as a Source Data file.

# Supplementary figure 8

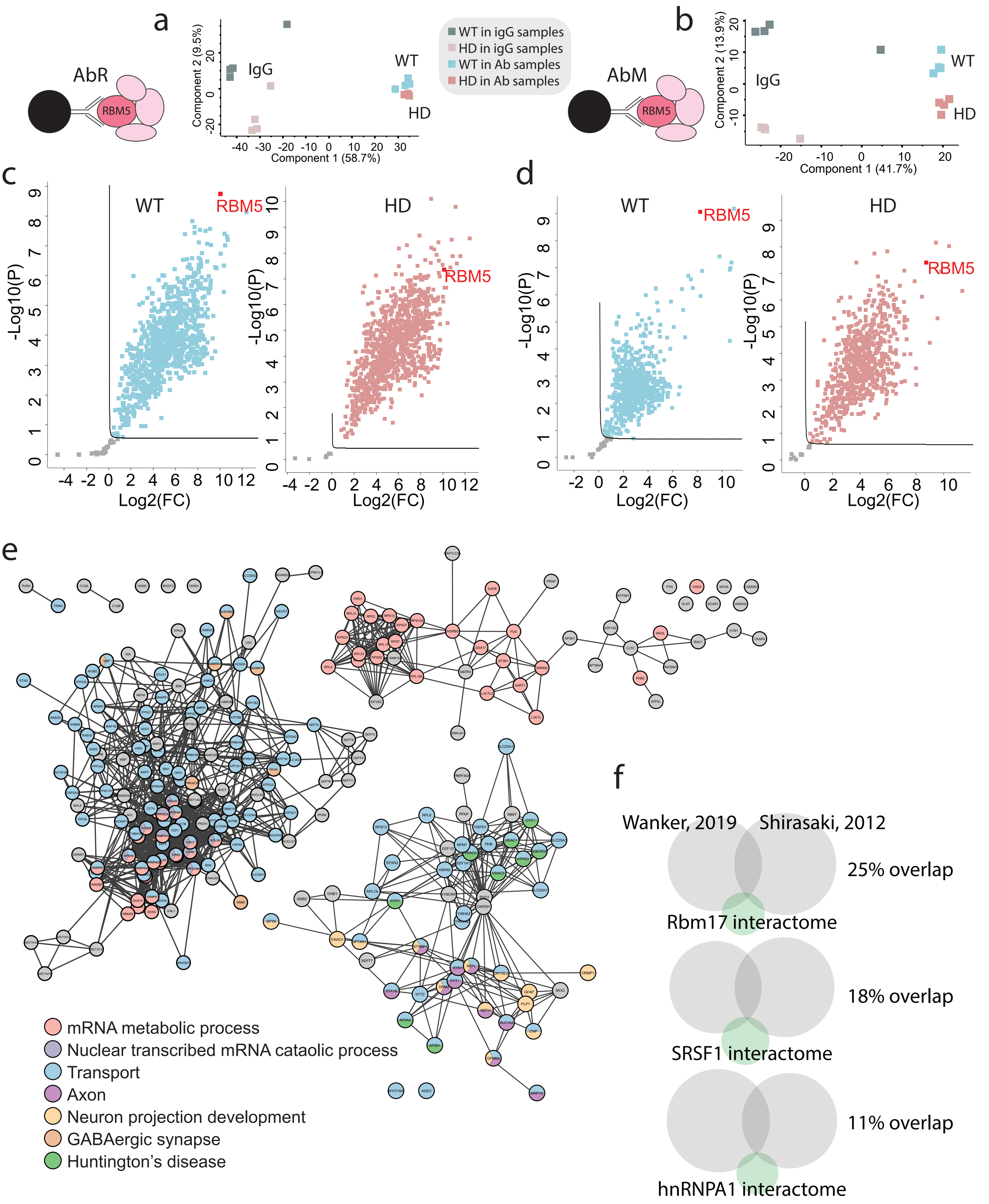

**Supplementary Figure 8. RBM5 Co-IP. (A-B)** PCA plot of RBM5 Co-IP samples immunoprecipitated with AbR (A) or AbM (B) from WT and HD mouse brain samples and the negative no-Ab control. **(C-D)** Volcano plot of the Co-IP samples with AbR (C) or AbM (D) compared to the no antibody control; threshold for significant enrichment in AbM or AbR Co-IPs compared to no antibody samples is marked with a black line and enriched proteins are marked with blue (WT) or red (HD). The bait – RBM5 – is highlighted in red. Significance was calculated using a one-sided t-test with multiple-testing correction. **(E)** Network representation of RBM5 protein interactors identified in the brain in this study and the HTT interactome from two publications. Overlap between our RBM5 interactome and previously published HTT interactomes (top left & right)[5,6] or both studies (bottom right). Colours of the protein nodes signify GO term annotations. **(F)** Overlaps between the two HTT interactome publications and previously identified interactomes for other splicing factors [7,8]. On the right, the percent of protein in the splicing factor interactomes that are also identified in the HTT interactomes is noted.

# Supplementary figure 9

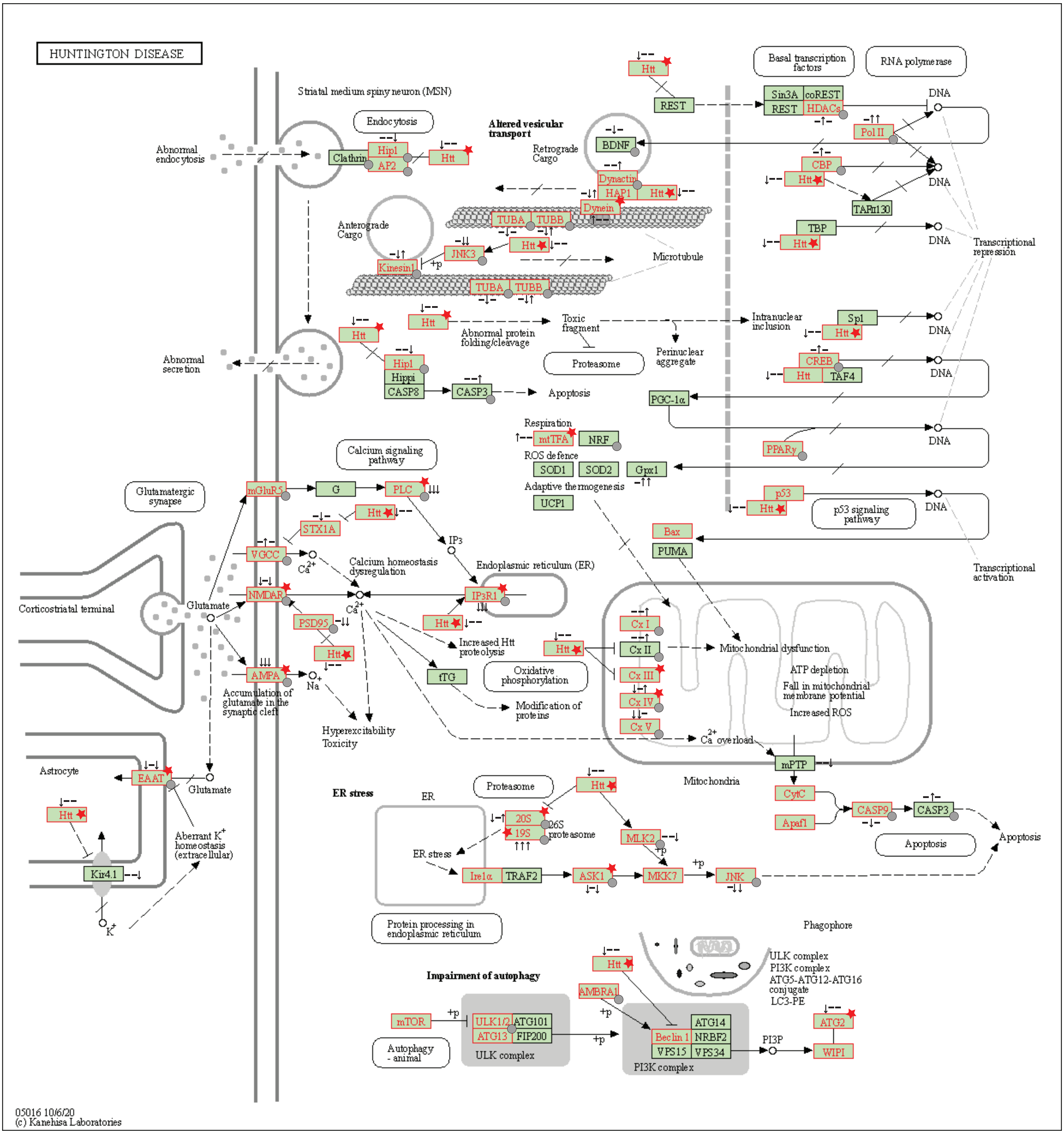


Text All RBM5 CLIP targets ★ Bound differentially by RBM5 ● Changes in splicing detected in HD

Arrows signify regulation in HD in the following order: 1) RBM5 binding 2) transcriptome 3) proteome.
Down (↓) Up (↑) No change (–)

**Supplementary figure 9. Huntington's disease KEGG pathway, highlighting RBM5 RNA-interactors and regulation in HD.** A compilation of the RBM5-RNA interactions and their changes in HD, transcriptome and splicing changes identified in this study and in a previous proteome study [3] for genes found in the Huntington's disease KEGG pathway. Genes highlighted in red are RBM5-CLIP targets in the brain. Red stars signify transcripts bound differentially by RBM5 and grey circles splicing changes in HD. The arrows next to each gene show direction of change in HD in the RBM5-CLIP data, transcriptome or proteome data, in this order (with an arrow or "-" for no change).

Supplementary references


1 Sharma, K. *et al.* Cell type- and brain region-resolved mouse brain proteome. *Nat Neurosci* **18**, 1819-1831, doi:10.1038/nn.4160 (2015).

2 Bekker-Jensen, D. B. *et al.* An Optimized Shotgun Strategy for the Rapid Generation of Comprehensive Human Proteomes. *Cell Syst* **4**, 587-599 e584, doi:10.1016/j.cels.2017.05.009 (2017).

3 Skotte, N. H. *et al.* Integrative Characterization of the R6/2 Mouse Model of Huntington's Disease Reveals Dysfunctional Astrocyte Metabolism. *Cell Rep* **23**, 2211-2224, doi:10.1016/j.celrep.2018.04.052 (2018).

4 Elorza, A. *et al.* Huntington's disease-specific mis-splicing unveils key effector genes and altered splicing factors. *Brain* **144**, 2009-2023, doi:10.1093/brain/awab087 (2021).

5 Shirasaki, D. I. *et al.* Network organization of the huntingtin proteomic interactome in mammalian brain. *Neuron* **75**, 41-57, doi:10.1016/j.neuron.2012.05.024 (2012).

6 Wanker, E. E., Ast, A., Schindler, F., Trepte, P. & Schnoegl, S. The pathobiology of perturbed mutant huntingtin protein-protein interactions in Huntington's disease. *J Neurochem* **151**, 507-519, doi:10.1111/jnc.14853 (2019).

7 De Maio, A. *et al.* RBM17 Interacts with U2SURP and CHERP to Regulate Expression and Splicing of RNA-Processing Proteins. *Cell Rep* **25**, 726-736 e727, doi:10.1016/j.celrep.2018.09.041 (2018).

8 Akerman, M. *et al.* Differential connectivity of splicing activators and repressors to the human spliceosome. *Genome Biol* **16**, 119, doi:10.1186/s13059-015-0682-5 (2015).
