# Supplementary material for: Characterising the RNA-binding protein atlas of the mammalian brain uncovers RBM5 misregulation in mouse models of Huntington’s disease
Source: Nat Commun. 2023 Jul 19;14:4348. doi: 10.1038/s41467-023-39936-x (PMC10356804; doi:10.1038/s41467-023-39936-x)
Supplement: Supplementary file 2 — Description of Additional Supplementary Files [file 41467_2023_39936_MOESM2_ESM.docx]

Description of Additional Supplementary Files:

File name: Supplementary data 1

Description: List of peptides identified as RNA binding in this study. Overlap with proteins identified in global RNA-interactome studies from mouse and human cell lines. Enrichment of domains identified among peptides identified in this study versus a brain proteome study.

File name: Supplementary data 2

Description: List of peptides and proteins identified as RNA binding from WT and HD mouse brain samples and comparison of peptide intensities between HD and WT samples.

File name: Supplementary data 3

Description: Rbm5 RNA-targets identified by CLIP analysis in WT and HD mouse brain tissue; list of clusters bound differentially by Rbm5 in HD.

File name: Supplementary data 4

Description: RNA-seq data from HD and WT mouse brain; list of transcripts, exons and introns with significantly different expression levels in HD.

File name: Supplementary data 5

Description: BAM files covering the *Rbm5* locus of the WT and HD mice used for the RNA-Seq analysis. The zip file contains the BAM files for this locus for 3 individual WT and 3 individual R6/2 mouse brains (indicated as HD).

File name: Supplementary data 6

Description: List of proteins identified as Rbm5 interactors in WT and HD tissue with two different antibodies.
