# Supplementary material for: Characterising the RNA-binding protein atlas of the mammalian brain uncovers RBM5 misregulation in mouse models of Huntington’s disease
Source: Nat Commun. 2023 Jul 19;14:4348. doi: 10.1038/s41467-023-39936-x (PMC10356804; doi:10.1038/s41467-023-39936-x)
Supplement: Supplementary file 9 — Reporting Summary [file 41467_2023_39936_MOESM9_ESM.pdf]

nature portfolio

Corresponding author(s): Meeli Mullari, Michael L. Nielsen

Last updated by author(s): Jun 28, 2023

Reporting Summary

Nature Portfolio wishes to improve the reproducibility of the work that we publish. This form provides structure for consistency and transparency in reporting. For further information on Nature Portfolio policies, see our Editorial Policies and the Editorial Policy Checklist.

Statistics

For all statistical analyses, confirm that the following items are present in the figure legend, table legend, main text, or Methods section.

n/a	Confirmed	
☐	☒	The exact sample size (*n*) for each experimental group/condition, given as a discrete number and unit of measurement
☐	☒	A statement on whether measurements were taken from distinct samples or whether the same sample was measured repeatedly
☐	☒	The statistical test(s) used AND whether they are one- or two-sided<br>*Only common tests should be described solely by name; describe more complex techniques in the Methods section.*
☒	☐	A description of all covariates tested
☐	☒	A description of any assumptions or corrections, such as tests of normality and adjustment for multiple comparisons
☐	☒	A full description of the statistical parameters including central tendency (e.g. means) or other basic estimates (e.g. regression coefficient) AND variation (e.g. standard deviation) or associated estimates of uncertainty (e.g. confidence intervals)
☐	☒	For null hypothesis testing, the test statistic (e.g. *F*, *t*, *r*) with confidence intervals, effect sizes, degrees of freedom and *P* value noted<br>*Give P values as exact values whenever suitable.*
☒	☐	For Bayesian analysis, information on the choice of priors and Markov chain Monte Carlo settings
☒	☐	For hierarchical and complex designs, identification of the appropriate level for tests and full reporting of outcomes
☐	☒	Estimates of effect sizes (e.g. Cohen's *d*, Pearson's *r*), indicating how they were calculated

Our web collection on statistics for biologists contains articles on many of the points above.

Software and code

Policy information about availability of computer code

Data collection	No custom software were used, MS data and RNA sequencing data were collected with the software provided with the instruments.
Data analysis	No custom software was used. All software used in the manuscript is publicly available and described in the methods. Software used: MaxQuant v1.4.1.1 and v1.5.0.38, Perseus v1.6.1.2, SeqMonq v1.47.1, ImageJ v1.52a, Cytoscape v3.8.2, GeneCodis98 and PANTHER version 11 and Prism version 8 were used in this study.

For manuscripts utilizing custom algorithms or software that are central to the research but not yet described in published literature, software must be made available to editors and reviewers. We strongly encourage code deposition in a community repository (e.g. GitHub). See the Nature Portfolio guidelines for submitting code & software for further information.

Data

Policy information about availability of data

All manuscripts must include a data availability statement. This statement should provide the following information, where applicable:

- Accession codes, unique identifiers, or web links for publicly available datasets
- A description of any restrictions on data availability
- For clinical datasets or third party data, please ensure that the statement adheres to our policy

The mass spectrometry proteomics data generated in this study have been deposited to the ProteomeXchange Consortium via the PRIDE partner repository with the dataset identifier PXD029227 [http://www.ebi.ac.uk/pride/archive/projects/PXD029227].
CLIP-seq and RNA-seq data can be accessed via the following GEO reviewer token: GSE187445 [https://www.ncbi.nlm.nih.gov/geo/query/acc.cgi?acc=GSE187445].
All other data generated in this study are provided in the Supplementary Information/Source Data file. Source data are provided with this paper.

Field-specific reporting

Please select the one below that is the best fit for your research. If you are not sure, read the appropriate sections before making your selection.

- [x] Life sciences
- [ ] Behavioural & social sciences
- [ ] Ecological, evolutionary & environmental sciences

For a reference copy of the document with all sections, see nature.com/documents/nr-reporting-summary-flat.pdf

Life sciences study design

All studies must disclose on these points even when the disclosure is negative.

Sample size	Samples-size calculation was not performed. All MS and Western blot analysis was performed in biological quadruplicates using individual brain samples derived from four individual mouse litter-mates. This choice is motivated by our previous experiments with requirements for label-free quantification and this approach was successfully applied in the paper introducing the pCLAP methodology (Mullari et. al, 2017). All RNA sequencing, Rbm5-CLIP and PCR analysis was done in biological triplicates (using individual brain samples derived from individual mouse litter-mates). CLIP analysis on were performed on individually cultured and transfected replicates. The sample sizes were chosen based about what is required to perform statistical tests consistent with previous publications, and are common practice in the field and our own experience (Fossat et. al, 2023).<br><br>References:<br>Mullari, Meeli, et al. "Specifying RNA-binding regions in proteins by peptide cross-linking and affinity purification." Journal of proteome research 16.8 (2017): 2762-2772.<br>Fossat, Nicolas, et al. "Identification of the viral and cellular microRNA interactomes during SARS-CoV-2 infection." Cell reports 42.4 (2023).
Data exclusions	One replicate from each genotype from the brain-pCLAP experiment done for the comparison of HD and WT samples (data presented in figure 3, supplementary table 2) were excluded due to an unforeseen and unfortunate RNA-degradation in the sample. This is a predefined condition for exclusion and could be determined by the extremely low total sample intensity based on experience with previous experiments.
Replication	All MS and WB analysis were done in biological quadruplicates, each sample represents an individual mouse brain derived from individual littermates. All RNA-seq, Rbm5-CLIP analysis and PCR amplification experiments were similarly performed in biological triplicates, each sample represents an individual mouse brain from individual littermates. CLIP from cells was done on independent cell culture triplicates. All the replicates were successful (excluding the 2 MS replicates mentioned under data exclusion) and all the data is shown in the manuscript.
Randomization	Samples were not divided into experimental groups, all replicates for all individual experiments were simultaneously prepared, handled, and statistically processed while taking multiple-hypotheses testing into account.
Blinding	All samples relating to each experiment were handled simultaneously. During handling, all samples were numbered and processed in random order to avoid introduction of bias into the samples. During MS data acquisition, samples were clearly labeled (and thus not blinded), which is important to MS experimental design. All data analysis was performed with unbiased software, in an unsupervised manner, and therefore blinding is not applicable in this context. All MS data is publicly available and may be re-processed and investigated by any external party.

Reporting for specific materials, systems and methods

We require information from authors about some types of materials, experimental systems and methods used in many studies. Here, indicate whether each material, system or method listed is relevant to your study. If you are not sure if a list item applies to your research, read the appropriate section before selecting a response.

Materials & experimental systems

n/a	Involved in the study
☐	☒ Antibodies
☐	☒ Eukaryotic cell lines
☒	☐ Palaeontology and archaeology
☐	☒ Animals and other organisms
☒	☐ Human research participants
☒	☐ Clinical data
☒	☐ Dual use research of concern

Methods

n/a	Involved in the study
☒	☐ ChIP-seq
☒	☐ Flow cytometry
☒	☐ MRI-based neuroimaging

Antibodies

Antibodies used	The following primary antibodies were used for immunoblot analysis in this study, and diluted at 1:1,000. For western-blotting, CLIP and GDP-PD of GFP-tagged candidate RBPs the anti-GFP mouse antibody (11814460001; Roche) was used. For Western-blotting, Rbm5-CLIP analysis and RBM5-CoIP the rabbit polyclonal anti-RBM5 antibody (HPA018011; Merck/Sigma) and mouse monoclonal anti-LUCA15 (200 µg/mL, G-2, sc-515419, Santa Cruz) were used. The Rabbit polyclonal to GAPDH (ab9485) antibody was used as a

	loading control.
Validation	Validation of the antibodies is stated on the supplier's website:<br>anti-GAPDH (ab9485): https://www.abcam.com/gapdh-antibody-loading-control-ab9485.html<br>anti-LUCA15 (sc-515419): https://datasheets.scbt.com/sc-515419.pdf<br>anti-GFP (11814460001): https://www.sigmaaldrich.com/deepweb/assets/sigmaaldrich/product/documents/270/763/11814460001.pdf<br>Validation for the HPA018011 anti-RBM5 antibody, validations is stated on the human protein atlas website: https://www.atlasantibodies.com/products/antibodies/primary-antibodies/triple-a-polyclonals/rbm5-antibody-hpa018011/

Eukaryotic cell lines

Policy information about cell lines

Cell line source(s)	HEK293T cells (CRL-3216, female), were acquired via the American Type Culture Collection and hESC (RC17 from Roslin Cells, hPSCreg RCe021-A)
Authentication	Cells were not routinely authenticated.
Mycoplasma contamination	All cells were tested for mycoplasma regularly and tested negative.
Commonly misidentified lines (See ICLAC register)	No commonly misidentified cell lines were used in this study.

Animals and other organisms

Policy information about studies involving animals; ARRIVE guidelines recommended for reporting animal research

Laboratory animals	R6/2, transgenic for exon 1 of the human HTT gene 40, originated from The Jackson Laboratory (Bar Harbor, Maine, USA) and were maintained by backcrossing males to CBA/j3B6 females (Taconic, Denmark). The mice were kept under specific pathogen-free conditions at a 12 hr light/12 hr darkness cycle in standard polystyrene cages with ad libitum access to standard chow and water. Mice were housed at 21C (20-24C temperature range) and at a humidity of 55 ± 10%. 12-week-old male mice were used for all experiments.
Wild animals	The study did not involve wild animals.
Field-collected samples	This study did not involve samples collected from the field.
Ethics oversight	Experiments were performed in accordance with the Danish Animal Experiments Inspectorate's guidelines (permit 2007/561-1345), the Danish Working Environment Authority (permit 20070033239/4), and European Commission Directive 86/609/EEC for animal experiments.

Note that full information on the approval of the study protocol must also be provided in the manuscript.
